# The Impact of Comorbidities among Ethnic Minorities on COVID-19 Severity and Mortality in Canada and the USA: A Scoping Review

**DOI:** 10.3390/idr16030030

**Published:** 2024-04-23

**Authors:** Christina Mac, Kylem Cheung, Tala Alzoubi, Can Atacan, Hibah Sehar, Shefali Liyanage, Bara’ Abdallah AlShurman, Zahid Ahmad Butt

**Affiliations:** 1 School of Public Health Sciences, University of Waterloo, Waterloo, ON N2L 3G1, Canada; christina.mac@uwaterloo.ca (C.M.); t2alzoubi@uwaterloo.ca (T.A.); catacan@uwaterloo.ca (C.A.); hsehar@uwaterloo.ca (H.S.); shefaliliyanage@gmail.com (S.L.); baalshurman@uwaterloo.ca (B.A.A.); 2 Department of Biochemistry and Biomedical Sciences, McMaster University, Hamilton, ON L8N 3Z5, Canada; cheunk33@mcmaster.ca

**Keywords:** COVID-19, adults, comorbidity, mortality, ethnic groups, minority groups

## Abstract

(1) Current literature on ethnic minorities, comorbidities, and COVID-19 tends to investigate these factors separately, leaving gaps in our understanding about their interactions. Our review seeks to identify a relationship between ethnicity, comorbidities, and severe COVID-19 outcomes (ICU admission and mortality). We hope to enhance our understanding of the various factors that exacerbate COVID-19 severity and mortality in ethnic minorities in Canada and the USA. (2) All articles were received from PubMed, Scopus, CINAHL, and Ovid EMBASE from November 2020 to June 2022. Included articles contain information regarding comorbidities among ethnic minorities in relation to COVID-19 severity and mortality. (3) A total of 59 articles were included that examined various ethnic groups, including Black/African American, Asian, Hispanic, White/Caucasian, and Indigenous people. We found that the most examined comorbidities were diabetes, hypertension, obesity, and chronic kidney disease. A total of 76.9% of the articles (40 out of 52) found a significant association between different races and COVID-19 mortality, whereas 21.2% of the articles (11 out of 52) did not. (4) COVID-19 ICU admissions and mortality affect various ethnic groups differently, with Black patients generally having the most adverse outcomes. These outcomes may also interact with sex and age, though more research is needed assessing these variables together with ethnicity.

## 1. Introduction

COVID-19 emerged in late 2019 and has since become a global pandemic, with over 672 million confirmed cases reported worldwide [1,2]. The virus is caused by the SARS-CoV-2 virus, which is spread from person to person through respiratory droplets or aerosols from coughing or sneezing [3]. Previous studies showed that older adults, essential workers, particularly those in healthcare settings, individuals in low-income and marginalized communities, and those with pre-existing medical conditions, including obesity, diabetes, lung/heart/kidney diseases, and weakened immune systems, are most at risk for severe illness from COVID-19 [4]. Some other studies have suggested that ethnic and racial minorities are also disproportionately and severely affected by COVID-19, but the relationship between ethnicity/race and COVID-19 outcomes remains controversial and is a matter of debate in the scientific community [5].

Race is defined as the categorization of individuals into distinct groups based on physical or social characteristics, whereas ethnicity is defined as belonging to or identifying as a group of people that share a common culture [6]. Ethnic minorities are defined as groups of individuals that have observable characteristics and cultures that differ from the majority in a population [7]. During the COVID-19 pandemic, research has been developed to gain a better understanding of the impact of COVID-19 among ethnic minorities compared to the majority [5].

Despite ongoing research, there remains an inconsistency in the results, with some studies finding a significant association between race/ethnicity and COVID-19 outcomes, while others do not. For instance, a systematic review by Mackey et al. found that African American populations suffer a larger number of COVID-19 deaths, as well as have a higher risk for hospitalization when compared with non-Hispanic White populations [8]. Another study found that these disparities in the outcomes may not be solely due to race [9]. Smedley (2003) suggests that comorbidities, ethnic health disparities, poverty, and access to healthcare may also contribute to these disparities in COVID-19 outcomes. This idea is supported by a review by Raharja et al., which found that differences in mortality between ethnicities were not significant after adjusting for age, sex, and comorbidities between Black, Asian, Hispanic, and White populations [10]. Similarly, the findings of ten cohort studies also did not support ethnicity as an independent risk factor for severe COVID-19 outcomes [10].

Looking at comorbidities alongside ethnicity and race may help with understanding how these patient characteristics are related to COVID-19 outcomes. Comorbidities are defined as the presence of two or more diseases or medical conditions in a patient [11]. Pre-existing comorbidities, such as chronic obstructive pulmonary disease (COPD) [12,13], chronic kidney disease (CKD) [14,15], cerebrovascular disease [15,16,17], cardiovascular disease [13,15,17], hypertension [13,14,15], and diabetes [13,14,15], have been found to be associated with increased severity and mortality of COVID-19. These previous findings highlight the importance of studying how comorbidities may mediate the relationship between race/ethnicity and COVID-19 outcomes.

The current literature on ethnic minorities, comorbidities, and COVID-19 severity and mortality tends to examine and focus on each factor separately, leaving gaps in our understanding of the interplay between these factors. Undoubtedly, these factors should be studied collectively to provide a holistic understanding of the risk factors for COVID-19 severity and mortality. To address these gaps, the purpose of our study is to examine which comorbidities are more prevalent in those who experience severe COVID-19 or mortality among adults of ethnic minorities in Canada and the USA. By examining the interaction between ethnicity, comorbidities, and severe COVID-19 outcomes, our primary research question seeks to identify a relationship between ethnicity, comorbidities, and severe COVID-19 outcomes (ICU admission and mortality). Furthermore, our secondary research question explores the impact of sociodemographic factors on COVID-19 severity and mortality in ethnic minorities. The overarching objective of this scoping review is to enhance our understanding of the various factors that exacerbate COVID-19 severity and mortality outcomes in ethnic minorities in Canada and the USA.

## 2. Materials and Methods

This review was conducted based on the methodological framework for scoping reviews outlined by Arksey and O’Malley [18].

### 2.1. Search Strategy

The following four databases were used for collecting articles for this scoping review: PubMed, Scopus, CINAHL, and Ovid EMBASE. Each search strategy contained MeSH terms, keywords, and CADTH COVID-19 search strings if applicable. As a result, included terms were related to COVID-19, severity and mortality, comorbidities, and ethnic minorities. For example, the search strategies generally included: (COVID-19 OR SARS-CoV-2 OR coronavirus OR COVID19 OR 2019ncov OR “coronavirus disease 2019” OR COV19) AND (sever* OR mortali* OR fatal* OR hospitaliz* OR hospitalis* OR “critical care” OR “intensive care”) AND (multimorbid* OR comorbid* OR co-morbid* OR “multiple non-communicable disease*” OR “multiple noncommunicable disease*” OR “multiple disease*” OR “multiple morbid*” OR “multiple chronic disease*” OR “multiple condition*” OR diabetes OR “diabetes mellitus” OR hypertension OR “cardiovascular disease*” OR “coronary heart disease*” OR cardiomyopath* OR “coronary artery disease*” OR “vascular disease*” OR “myocardial infraction*” OR “heart attack*” OR “heart failure” OR “heart disease*” OR arrhythmia* OR stroke* OR “respiratory disease*” OR “chronic obstructive pulmonary disease*” OR asthma OR “chronic kidney disease*” OR obesity OR “rheumatic disease*” OR arthritis OR “inflammatory bowel disease*” OR osteoarthritis OR osteoporosis OR “multiple sclerosis” OR “liver disease*” OR “kidney disease*” OR autoimmune OR “angina pectoris” OR “cardiac arrest”) AND (“ethnic minorit*” OR “minority group*” OR “racial minorit*” OR “ethnic minority group*” OR “people of colour” OR “African American*” OR black* OR hispanic* OR latin* OR “Mexican American*” OR “Asian American*” OR “Asian” OR “East Asian” OR “South Asian” OR “Southeast Asian” “Indigenous” OR “Native American*” OR “First Nations” OR Chinese OR Korean OR Japanese OR Vietnamese OR “racial disparit*” OR “ethnic disparit*”). Search results were also limited to the English language and dates of publication between 1 November 2020 to 26 June 2022.

### 2.2. Inclusion and Exclusion Criteria

Throughout the title/abstract screening and full-text review, the following inclusion and exclusion criteria were used to select the final articles for our scoping review. Firstly, the inclusion criteria consisted of peer-reviewed articles originating from Canada or the USA with a target population of adults 18 years old or above. Countries other than Canada or the USA were excluded from the study due to differences in ethnic minority groups. Original articles such as cross-sectional studies, case-control studies, cohort studies, randomized-controlled trials, and qualitative studies were included. As mentioned previously, these studies had to be published within November 2020 to June 2022 since this time frame pertains to the COVID-19 pandemic at the time of our study. The included articles had to examine COVID-19 severity, hospitalization, health outcomes, and/or mortality in relation to another disease. Additionally, these studies had to include health disparities among ethnic minorities. Lastly, mortality and intensive care unit (ICU) admissions were used as a measure of severe COVID-19 outcomes within our review. The term mortality refers to death of patients associated with a COVID-19 infection, while ICU admissions refers to patients who were admitted to the ICU within the hospital. Hospitalization due to COVID-19 infection was not considered the same outcome as ICU admission for the purpose of our study.

Articles were excluded from our scoping review if they met any of the following criteria: (1) They focused on pregnancy, cancer, genetic diseases/factors, mental health conditions, or communicable diseases; (2) The article did not mention COVID-19; (3) They focused on treatment methods, including alternative methods; (4) They were not peer-reviewed, such as conference papers, editorials, letters, or newspaper articles; (5) They were not written in English or not available in full text; or (6) They did not include either mortality or ICU admissions as outcomes.

### 2.3. Screening and Data Extraction

The title/abstract screening and full-text review were conducted on Covidence (Veritas Health Innovation, Melbourne, Australia) https://www.covidence.org/, a systematic review management software. All articles received from the search strategies conducted in PubMed, Scopus, CINAHL, and Ovid EMBASE were imported into Covidence for screening. The title/abstract screening and full-text review were conducted by C.M., K.C., C.A., T.A., B.A.A., and S.L. Any conflicts/disagreements were resolved by C.M.

Studies that were included in the final scoping review were extracted in a table. Information collected included: first author, year of publication, country and location, type of study, sample size, study population, ethnicity group(s) examined, comorbidities/conditions examined, comparisons of comorbidity prevalence between ethnicity groups, outcomes (ICU admissions or mortality) and rates, and other findings (Supplementary Table S1). A second table (Supplementary Table S2) was created to track information on sociodemographic factors in relation to our secondary research question. The data extraction process was completed by C.M., K.C., T.A., H.S., and C.A. The entire process was guided and monitored by Z.A.B. and B.A.A.

## 3. Results

### 3.1. PRISMA

**Figure 1:**
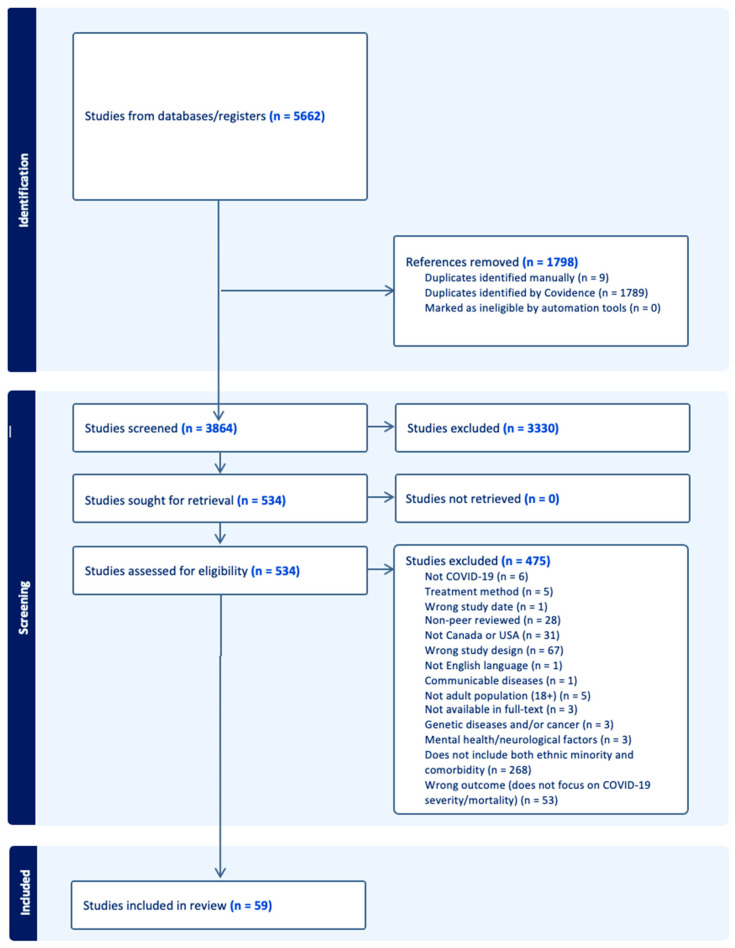
Preferred Reporting Items for Systematic Reviews and Meta-Analyses extension for Scoping Reviews (PRISMA-ScR) flowchart of article extraction from the literature search.

Based on our PRISMA flowchart (Figure 1), a total of 5662 studies were identified through database searches. From the 5662 studies, 1670 were collected from PubMed, 2703 from Ovid, 732 from Scopus, and 557 from CINAHL. After importing to Covidence, 1798 duplicates were removed, leading to 3864 studies being included in the screening process. In the tile abstract screening, 3330 were excluded as they did not meet our inclusion and exclusion criteria. In the full-text screening, 534 articles were reviewed, 475 of which were excluded based on the exclusion criteria as shown in Figure 1. As a result, a total of 59 studies were included in our review.

### 3.2. Descriptive Analysis

All the final 59 studies were conducted in the United States; none were conducted in Canada. Of these 59 studies, 18 (30.5%) were conducted across the USA [19,20,21,22,23,24,25,26,27,28,29,30,31,32,33,34,35,36]. Eight studies were conducted in Michigan [37,38,39,40,41,42,43,44], six in New York [45,46,47,48,49,50], four in Atlanta [51,52,53,54], and four in California [55,56,57,58]. The remaining studies were divided as follows: one in three states (California, Oregon, and Washington) [59], one in Massachusetts [60], one in Tennessee [61], two in Wisconsin [62,63], one in Mississippi [64], one in Cleveland [65], one in Pennsylvania [66], two in Louisiana [67,68], one in the Midwest [69], one near the Mexico border [70], one in New Orleans [71], one in Georgia [72], one in Northeast Ohio and South Florida [73], one in Milwaukee and Southeast Wisconsin [74], one in Florida [75], one in Missouri [76], and one in Illinois [77] (Table 1).

With respect to the study design, the selected 59 studies can be categorized into studies that were retrospective in nature, cross-sectional in nature, association studies, case-control studies, and cohort studies. There were 45 studies that were retrospective in nature, including observational studies [19,20,22,26,27,29,30,31,32,33,34,35,36,38,39,40,41,43,44,46,47,49,51,52,53,57,58,59,60,61,62,64,68,70,71,72,76]. Additionally, there was one association study [37], seven cohort studies [21,24,25,28,45,54,73], five cross-sectional studies [23,55,63,74,77], and one case-control study [48] (Table 1).

Overall, the following races/ethnicities were examined in our included studies: Black/African American, Asian, Hispanic, White/Caucasian, and Indigenous people. Firstly, 58 studies included Black/African American patients [19,20,21,22,23,24,25,26,27,28,29,30,31,32,33,34,35,36,37,38,39,40,41,42,43,44,45,46,47,48,49,50,51,52,53,54,56,57,58,59,60,61,62,63,64,65,66,67,68,69,70,71,72,73,74,75,76,77], 19 studies included Asian patients [25,29,31,32,33,36,44,45,47,48,49,53,57,58,59,64,65,72,77], 34 studies included Hispanic patients [19,24,27,31,32,35,36,44,45,46,48,49,50,52,53,55,56,57,58,59,60,61,65,66,70,73,74,75,76,77], 56 studies included White/Caucasian patients [19,20,21,22,23,24,25,26,27,28,29,30,31,32,33,34,35,36,37,39,40,41,43,44,45,46,48,49,50,51,52,53,54,55,56,57,58,59,60,61,62,63,64,65,66,67,68,69,71,72,73,74,75,76,77], and five studies included Indigenous patients [33,36,63,64,65] (Table 1).

**Table 1:** Summary of descriptive analysis results.

Location	# of Studies	Study Design	# of Studies	Race/Ethnicities	# of Studies
Across the USA [19,20,21,22,23,24,25,26,27,28,29,30,31,32,33,34,35,36]	18	Retrospective [19,20,22,26,27,29,30,31,32,33,34,35,36,38,39,40,41,43,44,46,47,49,51,52,53,57,58,59,60,61,62,64,68,70,71,72,76]	45	Black/African American [19,20,21,22,23,24,25,26,27,28,29,30,31,32,33,34,35,36,37,38,39,40,41,42,43,44,45,46,47,48,49,50,51,52,53,54,56,57,58,59,60,61,62,63,64,65,66,67,68,69,70,71,72,73,74,75,76,77]	58
Michigan [37,38,39,40,41,42,43,44]	8	Cohort [21,24,25,28,45,54,73]	7	White/Caucasian [9,10,11,12,13,14,15,16,17,18,19,20,21,22,23,24,25,26,27,28,29,30,31,32,33,34,35,36,37,39,40,41,43,44,45,46,48,49,50,51,52,53,54,55,56,57,58,59,60,61,62,63,64,65,66,67,68,69,71,72,73,74,75,76,77]	56
New York [45,46,47,48,49,50]	6	Cross-sectional [23,55,63,74,77]	5	Hispanic [19,24,27,31,32,35,36,44,45,46,48,49,50,52,53,55,56,57,58,59,60,61,65,66,70,73,74,75,76,77]	34
Atlanta [51,52,53,54]	4	Case-control [48]	1	Asian [25,29,31,32,33,36,44,45,47,48,49,53,57,58,59,64,65,72,77]	19
California [55,56,57,58]	4	Association [37]	1	Indigenous [33,36,63,64,65]	5
Other [59,60,61,62,63,64,65,66,67,68,69,70,71,72,73,74,75,76,77]	20				

### 3.3. Comorbidities

The most examined comorbidities were diabetes, hypertension, obesity, and kidney disease, with the least examined being pulmonary fibrosis, gastroesophageal reflux disorder, nephritis, fluid and electrolyte disorders, and influenza. Out of the 59 studies included, 52 studies examined diabetes, 42 studies examined hypertension, 28 studies examined obesity, and 31 studies examined kidney disease as comorbidities of COVID-19.

According to 34 studies included, Black patients had a higher prevalence of several comorbidities, such as diabetes, hypertension, obesity, and chronic kidney disease, than White patients [65,68]. A retrospective observational study by Krishnamoorthy et al. found that Black patients under the age of 65 years old had a lower prevalence of diabetes than those aged 65 years and older (34.9% vs. 46.9%). In contrast, they concluded that, in White patients, there was no association between the prevalence of diabetes in those aged 65 years old and younger in comparison to those aged 65 years and above (27% vs. 34.3%) [43]. Patients with a higher prevalence of obesity had increased odds of ICU admissions [38]. In several studies, White patients presented with a higher prevalence of chronic obstructive pulmonary disease (COPD) [22,29,30,53,69,72,73], coronary artery disease [29,53,72,73], and congestive heart failure [53,73], while Hispanic patients were less likely to present with these comorbidities [24]. In comparison, Asian patients had the lowest prevalence of chronic pulmonary disease, diabetes, obesity, and liver disease [36] (Table 2).

In contrast, a retrospective cohort study by Page-Wilson et al. concluded that race and ethnicity did not impact COVID-19-related mortality [47].

### 3.4. Mortality and ICU Admissions

Out of the 59 articles, 52 of the articles mentioned mortality [20,21,23,24,25,26,27,28,29,30,31,32,33,34,35,36,37,38,39,40,41,42,43,44,45,46,47,48,49,50,51,52,54,55,56,57,59,61,63,64,65,66,67,68,69,70,71,72,73,74,75,76,77], 32 mentioned ICU admissions [19,21,23,24,25,26,30,37,38,39,40,41,45,49,52,53,54,55,56,58,60,62,63,65,66,68,69,70,71,73,74,75], and 26 that mentioned both [21,23,24,25,26,30,37,38,39,40,41,45,49,52,54,55,56,63,65,66,67,68,69,70,73].

#### 3.4.1. Mortality

With regards to racial differences and COVID-19 mortality, 76.9% of the articles (40 out of 52) found a significant association of different races and COVID-19 mortality, whereas 21.2% of the articles (11 out of 52) did not [41,45,47,51,54,55,56,57,65,68,69]. The races compared were between Black and all other race categories (White, Asian, American Indian/Alaska Native, Native Hawaiian/Pacific Islander, and multiple races) [51,54], Black and White [65], non-Hispanic White, non-Hispanic Black, Hispanic and Asian patients [45,57,77], non-Hispanic Black and non-Hispanic White [41,68,69], Hispanic and non-Hispanic patients [55,56], Black and Asian [47], Non-Hispanic Black, Non-Hispanic White, and Hispanic [74]. Previous studies reported higher COVID-19-related mortality rates in ethnic minorities including, but not limited to, Black, Hispanic, and Asian races when compared to White patients [20,24,27,28,30,32,34,35,37,44,49,59,61,63,64,67] (Table 3).

More specifically, the risk of COVID-19 mortality was 1.3-times higher in Black than White patients [67]. Another study showed an increasing risk of 3.5 times in COVID-19 mortality when comparing the Black and White patients who had comorbidities and were within the same age categories [44]. In addition to this Kalyanaraman et al. found a 29% vs. 12% mortality rate when comparing Black patients with White patients in the USA, New York City [46], whereas Navar et al. found a 22.7% vs. 20.8% COVID-19 mortality rate when comparing Black patients with White patients [33]. Furthermore, a positive correlation exists between COVID-19 mortality rate and the proportions of Black individuals in a county, as reported by Millett et al. [20]. Other races, such as Hispanic patients, were also prone to having a higher mortality rate when compared to White patients [24,27,28,35,49,59] (Table 3).

Conversely, other studies highlighted an opposite trend, where White patients had a higher COVID-19 mortality rate than Black patients [25,27,32,58,60] and Hispanic patients [46]. The differences in COVID-19 mortality rate among White patients compared to Black patients were inconsistent among previous studies. For instance, in the USA, across 92 hospitals, the authors reported a 23.1% vs. 19.2% COVID-19 mortality rate in White patients compared to Black patients [21], respectively, while in the USA, New York, 47% vs. 32% was reported [46]. On the other hand, no significant association was observed between COVID-19 mortality rates and Hispanic and non-Hispanic patients [56] (Table 3).

Another article found a higher mortality rate in American Indians at 41% compared to White patients at 22.6% [64] (Table 3).

**Table 3:** Summary of mortality findings based on ethnicity.

Overall Results	Key Findings
Higher COVID-19 mortality in Black patients than White patients.	1. The risk of COVID-19 mortality was 1.3 times higher in Black than White patients [67] 2. When comparing Black and White patients who have comorbidities within the same age categories, there is an increasing risk of 3.5 times in mortality [44] 3. 29% vs. 12% COVID-19 mortality rate when comparing Black and White patients respectively [46] 4. 22.7% vs. 20.8% COVID-19 mortality rate when comparing Blalck and white patients respectively [33] 5. A positive correlation exists between COVID-19 mortality rate and the proportions of Black individuals in a county [20]
Higher COVID-19 mortality in White patients than Black patients.	1. 23.1% vs. 19.2% COVID-19 mortality in White patients compared to Black patients [21] 2. 47% vs. 32% COVID-19 mortality in White patients compared to Black patients [46]
Higher COVID-19 mortality in White patients than Hispanic patients.	1. 47% vs. 32% COVID-19 mortality in White patients compared to Hispanic patients [46]
Higher COVID-19 mortality in American Indian patients than White patients.	1. 41% vs. 22.6% COVID-19 mortality in American Indian patients compared to White patients [64]
Higher mortality rate in Hispanic vs. other patients.	1. 6.3% vs. 4.5% mortality rate when comparing Hispanic and White patients respectively [74] 2. 11.9% vs. 26.3% 30-day mortality in Hispanic patients compared to non-Hispanic patients [77]

#### 3.4.2. ICU Admissions

Black patients were more likely to have obesity [38] and diabetes [55], and patients with obesity were found to have an increased chance of ICU admissions [38,47,55]. Black patients had higher ICU admission rates, 6.3% vs. 2.8% [40], 15.2% vs. 13.6% [66], 12.5% vs. 7.8% [74], and 32.8% vs. 22.4% [75], compared to White patients. Black patients were also found to have a higher ICU admission rate compared to other racial groups (20.2% vs. 17.2%), such as White, Native Hawaiian, Native American or Alaska Native, Asian, and unknown [63]. Latinx patients were also found to have a higher admission rate to the ICU when compared to White patients (39% vs. 30%) [60]. Hispanic and Latinx patients were observed to be more than twice as likely to experience ICU admissions than White patients [58]. Asians were also found in one study to have the highest rates of ICU admissions when compared to other races, such as Black, White, Hispanic, and other [53]. ICU admissions between White patients and Black patients were 36.4% and 35.2%, respectively [21], while in another study, ICU admissions were lower for Hispanic patients than White patients [73]. Additional findings show that there are no significant differences in races regarding ICU admissions [41,55,56,65,69] (Table 4).

### 3.5. Sociodemographic Factors

All of the 59 studies included in our analysis reported sociodemographic factors directly associated with COVID-19 infections, mortality, and ICU admissions. The most common sociodemographic factors that were evaluated were age, sex, race/ethnicity, and insurance status, while the factors that were assessed less frequently were BMI, employment/occupation, smoking status, education, and household income. Age, sex, and race/ethnicity were highly associated with COVID-19 infections, mortality, and ICU admissions in the majority of the studies observed.

Thirteen studies demonstrated that older age, defined as those aged 50+ years, had higher rates of COVID-19 infection, ICU admission, and mortality [19,21,28,32,39,41,45,52,53,61,63,68]. A study stated the average age of confirmed cases among patients who were admitted to the ICU was 51 years old [72] in correlation to a study by Datta et al., which found that ICU admission rate changes were greater for older age groups (65+ years) [19]. Other studies concluded that the mean ages of patients that were hospitalized or died were lower among patients of a minority race/ethnicity, such as those who identified as African American and Hispanics/Latinx, in comparison to White or non-Hispanic patients [22,29,42,55,59,60,61,65]. According to a study by Zakaria et al., African American patients were significantly younger compared to White patients with a larger difference in those aged 60 years and younger, and this difference was seen in both females and males [42]. Another study quantified the difference by stating that hospitalized Hispanic and African American patients were significantly younger than non-Hispanic White patients with median ages of 57 and 60 years compared to 69 years, respectively [29]. In contrast, a retrospective study by Abate et al. concluded that patients who identified as African American or Caucasian had a higher mean age (63.3 and 67.2 years old, respectively) in comparison to patients who did not identify as African American or Caucasian (mean age of 57.3 years old) [69]. Two studies supporting this finding stated that hospitalized Caucasian patients were older, followed by Hispanic patients, Black patients, and then other races [73], with White patients having an average age of 71.8 years in comparison to Black patients with an average of 62.9 years of age [43] (Table 5).

Regarding sex as a factor, several studies found that the male sex was associated with higher risk of mortality, ICU admissions, screening positive, and overall adverse outcomes compared to female patients [25,28,32,39,41,45,52,53,61,62,68,74]. Ricardo et al. found that males had a higher percentage of a 28-day mortality, with 689 Hispanic males compared to 305 Hispanic females and 773 White males vs. 386 White females [24]. In comparison, Ghoneim et al. found that the overall incidence of COVID-19 infection was higher in females (60%) than in males (40%); however, across all counties, men with COVID-19 infections are at a higher risk of adverse outcomes and mortality [34]. Other studies showed that, among female patients, there was a higher proportion of African American females than White females, with the African American females presenting at a younger age [21,22,40,42,73]. Elbadawi et al. found that, among female patients with COVID-19 infections, 51.9% were Black, 43.9% were Caucasian, 41.4% were Hispanic, and other racial groups accounted for 29.3% [73]. Another study found that the group of African Americans had a higher proportion of female patients than White female patients with 53.4% and 45.7%, respectively [42] (Table 6).

In terms of race and ethnicity, the Black race was associated with an increased risk of COVID-19 susceptibility and adverse outcomes, such as high mortality [23,25,33,34,61,68]. Parker et al. found that patients who died from COVID-19 infections were more likely to identify as African American than case patients who survived the infection [61]. Escobar et al. found that the likelihood of a COVID-19 infection was significantly due to race (80.3%), with ICU admissions, in-hospital deaths, and total deaths among patients with COVID-19 infections throughout this study being higher among African American, Hispanic, and Asian patients [57]. With conflicting findings, a cohort study in Louisiana and Georgia [54], a retrospective cohort study in New York City [47], and a retrospective cohort study in Cleveland [65] concluded that race and ethnicity were not associated with COVID-19-related mortality. Shadyab et al. found that the rate of deaths and other poor outcomes were not significantly different between Hispanic and non-Hispanic patients with COVID-19 infections [56].

## 4. Discussion

This review was conducted to examine whether racial/ethnic minority status and having comorbidities are associated with COVID-19 ICU admissions and mortality outcomes. A total of 59 studies were included in this review. We observed that racial/ethnic minorities, especially Black patients, had increased COVID-19 mortality rates compared to White patients. Black and Hispanic patients generally had higher ICU admission rates than White patients, although some studies did not find differences. Black patients were more likely to have metabolic, nephrotic, and hypertensive comorbidities, while White patients were more likely to have respiratory and cardiovascular comorbidities, and Hispanic and Asian patients were less likely to have comorbidities. Black patients were often, on average, younger than White patients. Male patients were found to have a higher risk of COVID-19 mortality, though there were usually more female patients, especially among Black patients.

### 4.1. Differences in Mortality Rates

Our review determines that Black patients are more likely to have higher COVID-19-related mortality rates than White patients, which is consistent with other recently published literature [78]. Several plausible explanations can justify this finding. First, previous studies showed that Black patients were more likely to have multiple co- morbidities, such as obesity, diabetes, and chronic kidney disease, which may exacerbate COVID-19 presentations [68]. Second, Black patients were overrepresented in high-risk occupations that could not be shifted online, including frontline essential jobs [68]. Therefore, they had to travel to their work and had no choice to practice social distancing, which meant they were at higher risk of contracting COVID-19. Third, Black patients suffer from a number of social, economic, and health inequities that reflect their longstanding history of structural racism, such as living in poor and multigenerational housing, in neighborhoods with high crime rates, and in areas with water and air pollution. They also face barriers to accessing healthy food and to healthcare services, all of which lead to worse health outcomes [79].

### 4.2. Differences in ICU Admissions

In this review, some studies found that Black and Hispanic patients experienced higher ICU admission rates when compared with White patients, while other studies found no differences between the ethnic groups. A possible explanation for this inconsistency could be the contrasting study designs and the various confounding variables, such as age, that may not have been accounted for within the study.

In a study conducted by Pennington et al., Hispanic/Latino, Asian and “other” racial groups experienced a higher risk of ICU admissions when compared with White patients. However, the differences in ICU admissions between Black and White patients were minimal after adjusting for confounding variables. A limitation in this study, as mentioned earlier, is the inability to adjust for confounding variables, such as occupation, income, education, and access to healthcare [80].

Contrary to the previous study, Agyemang et al. found that, although Black and Hispanic patients were more likely to be hospitalized once infected, there was no difference in severe outcomes once hospitalized, including ICU admissions between the different races [81]. However, this study did find that Asian Americans had a higher ICU admission rate in the USA when compared to White patients.

The conflicting results found in these studies as well as this review can be attributed to confounding variables that may have been harder to quantify. One reason could be related to cultural factors, such as ethnic minority household sizes and multi-generational households, where the risk of contracting COVID is greater due to more individuals exposed to one another regularly. This could in turn lead to higher rates of infection, and thus, higher cases of ICU admissions. This would mean that the observed higher rates of ICU admissions would not be directly related to racial inequalities within the healthcare system, but perhaps simply a sociodemographic difference. As well, many other factors mentioned, such as a lack of insurance, language barriers, lack of a primary healthcare provider, and/or general access to healthcare, could also play a role in higher ICU admission rates being observed among ethnic minorities.

Overall, to come to a more conclusive result in ICU admission rates between ethnic minorities, further studies are warranted to account for a wide variety of factors, including specific sociodemographic/socioeconomic factors as well as comorbidities within ethnic minorities.

### 4.3. Differences in Comorbidities

In this review, the comorbidities that presented in patients with COVID-19 varied significantly according to race and ethnicity. In line with the findings of previous studies and reviews, our review highlighted the prevalence of certain comorbidities among different races and ethnicities in correlation to a COVID-19 infection. The review demonstrated that Black patients were more likely to have metabolic, nephrotic, and hypertensive comorbidities, such as diabetes, hypertension, chronic kidney disease, and obesity [19,22,28,30,31,34,35,42,43,45,52,64,69,71,72,73]. In contrast, White patients had a higher prevalence in respiratory and cardiovascular comorbidities, including coronary artery disease, congestive heart failure, and chronic obstructive pulmonary disease [23,28,30,41,43,54,56,73]. This differential prevalence of hypertension can be explained by examining the differences in environments and habits that Black patients versus White patients are exposed to and display. Several reasons deemed to be potential causes include access to good-quality healthcare and education, genetics, stress, social and environmental factors, diet, as well as the overall health behaviors demonstrated by both groups. For similar reasons, those who identified as Asian patients had the lowest prevalence of chronic pulmonary disease, diabetes, obesity, and liver disease [46,76], while Hispanic patients had a low prevalence of chronic pulmonary disease, coronary artery disease, and congestive heart failure in comparison to White patients [42,53,68]. Some demographic factors, such as education level, race, and socioeconomic status, can be accounted for when examining the differences across patient group races. Low educational levels, Black ethnicity, and low-income levels can explain the increase in the prevalence of comorbidities.

### 4.4. Differences in Age and Sex

Lastly, with respect to the sociodemographic factors, our review found that Black patients were, on average, younger than White patients. Additionally, male patients were found to have a higher risk of COVID-19 mortality, although there was a higher proportion of female patients among the Black patients. The current literature has conflicting conclusions regarding this topic. A cross-sectional study conducted by Elo et al. (2022) [82] found that Black and Hispanic patients had a higher mortality rate at all ages, except at the age of 85 years old and above. Furthermore, this study found that mortality changes were the worst among Black women compared to Black men at every age, and Black women had higher rates of mortality compared to Hispanic women [82]. These findings are in line with the findings from our study. From the comparison of previous studies, we hope to determine the factors that lead to these findings of disparities among ethnic minorities. Overall, the determinants of these results cannot be limited to sociodemographic factors. The findings from our study highlight the different impacts of various factors, such as comorbidities, ethnicity, and other sociodemographic factors, on disparities in COVID-19 severity and mortality.

### 4.5. Strengths and Limitations

Our analyses were based on data from across North America and included peer-reviewed published papers only across many papers from our research. An exhaustive search strategy was used involving a variety of keywords and MeSH terms in relation to our research topic, allowing a wide range of studies to be included in our final review, which provided us verifiable data from several papers. This review contained many studies from racially, ethnically, and geographically diverse populations in North America, allowing for a greater generalizability of our results. We acknowledge some limitations within our review where the results should be approached with these in mind. First, though our review yielded data from across Canada and the United States, most of the data were collected from papers and studies carried out in the USA with a restricted number of studies in Canada. These data allow for the generalizability of the results within North America, specifically the USA. With this, it is important to note that studies from Europe, Asia, Africa, and South America were not included, and this made the extrapolation of our results to these regions in the world challenging. Second, our review excluded studies with patients under the age of 18 years old. Therefore, the findings from this review could vary if they included children and adolescents. This exclusion limits the effects that a COVID-19 infection may have on the younger population in relation to racial and ethnic minorities, and these results may differ in comparison to the adult population that was included, preventing the application of our results to younger age groups. This also makes the generalization of our results difficult to this younger age group. Third, some of the articles included had a variety of categorizations for race, such as “unknown” or “other”, which could have led to incomplete or missing results in specific racial groups. The differences in the categorization of race might lead to misclassification bias when individuals are assigned to a different category than the one they should be in. Finally, non-English literature was excluded from this review, which may have resulted in the exclusion of relevant data and may have also reduced the generalizability of the results.

## 5. Conclusions

In conclusion, this review demonstrated that COVID-19 ICU admissions and mortality affect various racial/ethnic groups differently, with Black patients generally having the most adverse outcomes. Different racial/ethnic groups also had different types of comorbidities, which may have influenced the outcomes. These outcomes may also interact with sex and age, though more research is needed assessing these variables together with race/ethnicity. The differences in outcomes by race/ethnicity could be explained by lower socioeconomic status, working in high-exposure occupations, limited healthcare access, lack of a primary care provider, poor housing conditions, crowded households, lack of insurance, language barriers, lack of access to healthier foods, and lower educational attainment. Since all the studies were in the United States, future research on this topic should also be conducted in Canada. These findings suggest that the marginalization of racial/ethnic groups negatively affected their health during the COVID-19 pandemic. The findings from this review could be used to inform public health policies that aim to reduce inequities in COVID-19 outcomes among racial/ethnic groups with comorbidities, such as improving the healthcare access, insurance coverage, and housing conditions of racially and ethnically diverse, underserved communities.

## Figures and Tables

**Table 2:** Summary of comorbidities based on ethnicity results.

Racial/Ethnic Group	Key Findings
Black	1. Higher prevalence of diabetes, hypertension, obesity, and chronic kidney disease compared to White patients [65,68] 2. Black patients under the age of 65 had a lower prevalence of diabetes than those aged 65 and older (34.9% vs. 46.9%) [43]
White/Caucasian	1. No association between the prevalence of diabetes in those aged 65 and younger in comparison to those 65 years and older [43] 2. Higher prevalence of COPD [22,29,30,53,69,72,73] 3. Higher prevalence of coronary artery disease [29,53,72,73] 4. Higher prevalence of congestive heart failure [53,73]
Hispanic	1. Less likely to present with COPD, coronary artery disease and congestive heart failure [24]
Asian	1. Lowest prevalence of chronic pulmonary disease, diabetes, obesity, and liver disease [36]

**Table 4:** Summary of ICU admission findings based on ethnicity.

Race Comparison	Key Findings
Black vs. Others	1. 6.3% vs. 2.8% [40], 15.2% vs. 13.6% [66], 12.5% vs. 7.8% [74], and 32.8% vs. 22.4% [75] ICU admission rates in Black patients vs. White patients 2. Black patients also had higher ICU admission rates compared to other racial groups (20.2% vs. 17.2%) such as White, Native Hawaiian, Native American or Alaska Native, Asian, unknown [63] 3. Black patients had an ICU admission rate of 12.5% vs. Hispanic patients at 10.3% and White patients at 7.8% [74]
White vs. Others	1. 36.4% and 35.2% ICU admission rates in White patients vs. Black patients [21] 2. ICU admission rates lower in Hispanic patients than White patients [73]
Hispanic/Latinx vs. White	1. 39% vs. 30% ICU admission rates in Latinx patients vs. White patients [60] 2. Hispanic/Latinx patients were observed to be more than twice as likely to experience ICU admission [58]
Asian vs. Others	1. Highest rates of ICU admission compared to White, Black, Hispanic, and other races [53]
Others	1. Other findings showed no significant differences in races regarding ICU admissions [41,55,56,65,69]

**Table 5:** Summary of age findings based on ethnicity.

Race	Key Findings
Black/African American	1. African American patients were significantly younger compared to White patients, particularly those aged 60 years and younger [42] 2. Hospitalized African American patients were significantly younger than non-Hispanic White patients with median age of 60 compared to 69 [29] 3. Hospitalized patients who identified as African American or Caucasian had a higher mean age (63.3 and 67.2 respectively) in comparison to patients who did not identify as African American or Caucasian (mean age of 57.3) [69]
White	1. Hospitalized White patients were older, followed by Hispanic patients, Black patients and then other races [73] 2. Hospitalized White patients had an average age of 71.8 compared to Black patients, with an average age of 62.9 [43]
Hispanic	1. Hospitalized Hispanic patients were younger compared to non-Hispanic White patients with median age of 57 compared to 69 [29]. 2. The median age of ICU admission in Hispanic patients was significantly lower compared to non-Hispanic patients (56.6 years vs. 65.7 years)

**Table 6:** Summary of sex findings based on ethnicity.

Race/Race Comparison	Key Findings
Black vs. White	1. Among female patients, there was a higher proportion of African American females than White females with the African American females presenting at a younger age [21,22,40,42,73]. 2. Another study found that the group of African American race had a higher proportion of female patients than White female patients with 53.4% and 45.7% respectively [42].
White	1. Males had a higher percentage of 28-day mortality with 773 White males vs. 386 White females [24]
Hispanic	1. Males had a higher percentage of 28-day mortality with 689 Hispanic males compared to 305 Hispanic females [24]

## Data Availability

The data presented in this study are available on request from the corresponding author.

## Supplementary Materials

The following supporting information can be downloaded at: https://www.mdpi.com/article/10.3390/idr16030030/s1, Table S1: Data extraction table for the review, Table S2: Data extraction table including socio-demographic variables.
