# Supplementary material for: The Impact of Comorbidities among Ethnic Minorities on COVID-19 Severity and Mortality in Canada and the USA: A Scoping Review
Source: Infect Dis Rep. 2024 Apr 23;16(3):407–22. doi: 10.3390/idr16030030 (PMC11130838; doi:10.3390/idr16030030)
Supplement: Supplementary file 1 [file idr-16-00030-s001.zip › idr-2932604-supplementary.pdf]

Study #	First Author	Country	Type of Study	Sample Size	Ethnicity Group(s) Examined	Comorbidities/ Conditions Examined	Comparison of comorbidity prevalence between Ethnicity groups	Outcome (Mortality or ICU admissions)	Mortality or ICU Admission Rate
**1**	Wiley, Z. (2021) [51]	USA (Atlanta)	Retrospective cohort study	831<br>569 Black (68.5%)<br>32 Hispanic (3.9%)	•Black<br>•Non-Black	•Hypertension<br>•Diabetes<br>•Cardiovascular Disease<br>•Chronic Kidney Disease<br>•Dementia<br>•Chronic Pulmonary Disease<br>•End Stage Renal Disease<br>•Cancer<br>•HIV/AIDS	•Black patients had higher prevalence of obesity, diabetes, hypertension, dementia, HIV/AIDs, and chronic kidney disease	Mortality	•No significant findings were found in relation to rates of in-hospital mortality between Black and non-Black patients
**2**	Datta, B. K. (2022) [19]	USA	Retrospective analytical model		•Black<br>•White<br>•Hispanic<br>•Other	•Hypertension<br>•Diabetes<br>•Cardiovascular Disease	•Decline in rates of CVD would be smaller among Hispanics (1.1%)<br>•Same for White, Black, and other race categories (1.5%)<br>•Decline in rates of hypertension and diabetes among Black patients results in a decline in ICU admissions	ICU admissions	•There is an estimation that lower rates of hypertension would result in a lowered cumulative hospitalization and ICU admissions rates by more than 2.5%.<br>•Another estimation concludes that lower rates of diabetes and CVD would result in a lowered cumulative hospitalization rate by 0.6% and 1.4% respectively.<br>•These rates declining would be higher among the Black population and those who are classified as elder (aged 55 or older)
**3**	Salvatore, M. (2021) [37]	USA (Michigan)	Association Study	53,853	•Non-Hispanic Black<br>•Non-Hispanic White (White)<br>•African American (Black)	•Autoimmune Disease<br>•Kidney Disease<br>•Type 2 Diabetes<br>•Circulatory Disease<br>•Dementia<br>•Cancer<br>•Liver Disease	•Blacks have higher rates of autoimmune disease, kidney disease, type 2 diabetes, and circulatory disease<br>•White have higher rates of cancer (including skin cancer)	ICU admissions and Mortality	•Acidosis, pulmonary, and acute/chronic renal diseases demonstrated a significant relationship with hospitalization, ICU admissions and mortality in both White and Black races.<br>•Acute renal disease was extremely significant in mortality.<br>•The relation between circulatory system disorders, genitourinary

									diseases (stage I or II chronic kidney disease, p= 2.34 x 10-4) and COVID-19 mortality is more significant in Black patients than White patients.
**4**	Millett , G. A. (2020) [20]	USA	Retrospective study	N/A (looked at counties mainly)	•Black •White	•Hypertension •Diabetes	•Counties with more black residents have greater comorbidities, including diabetes	Mortality	•Having higher rates of Black residents in an area is related to higher rates of deaths and COVID-19 diagnoses in comparison to other areas
**5**	Olanipekun, T. (2022) [52]	USA (Atlanta)	Retrospective cohort study	181	•Black •White •Hispanic	•Diabetes •Hypertension •Chronic Liver Disease •Chronic Kidney Disease •Asthma •Coronary Artery Disease •Congestive Heart Failure	•African Americans and Hispanics have greater rates of multiple comorbidities compared to Whites	ICU admissions and Mortality	•Rates of mortality and ICU mortality were higher in patients with BMI ≥ 35 kg/m2 (43.4% vs. 32.2%, p < 0.01), prior cerebrovascular accident (CVA) compared to patients without CVA (62% vs. 32%, p < 0.01), CKD 3 and above (20.3% vs. 14.8%, = 0.02), in patients with hypertension compared to those without hypertension (71.2% vs. 59%, p <0.01) and several comorbidities (91.5% vs. 70.5%, p<0.01).
**6**	Suresh , S. (2021) [38]	USA (Michigan)	Retrospective cohort study	1983	•African American	•Diabetes •Hypertension •Sleep Apnea •Chronic Obstructive Pulmonary Disease •Asthma •Cardiovascular Disease •Obesity •Malignancy	•Patients with obesity were younger (P < 0.0001) compared to patients without obesity •Patients were more likely to be female compared to patients without obesity •Patients were more likely to be African American compared to patients without obesity •Patients with obesity had higher rates of ICU admissions	Primary outcomes were mortality, intensive care unit (ICU) admission, intubation, and 30-day readmission.	•Higher rates of ICU admissions were seen in patients with obesity

**7**	Hehar, J. (2022) [39]	USA (Michigan)	Retrospective cohort study	8751	•Black •White	•Diabetes •Chronic Kidney Disease (CKD) •Sleep Apnea •Chronic Obstructive Pulmonary Disease (COPD) •Coronary Artery Disease (CAD) •Stroke •Heart Failure (HF) •Cirrhosis •HIV Infection •Active malignancy	•Black patients had lower rates of death than White patients •Black had higher rates of diabetes, hypertension, CKD, sleep apnea, heart failure, HIV, active malignancy •Black patients were more likely to be overweight (BMI > 30.0 kg/m2)	Mortality and ICU admissions	•Black patients had lower rates of mortality than white patients •867 (9.9%) admitted to the ICU •753 (8.6%) passed away
**8**	Kabarr iti, R. (2020) [45]	USA (New York)	Cohort Study	9268	•Hispanic •Non-Hispanic Black •Non-Hispanic White (White) •Asian •Other	•Morbid Obesity (BMI > 35) •Cardiovascular Disease •Diabetes •Kidney Disease •Dementia	•Hispanic & non-Hispanic Black patients had higher rates of more than two comorbidities with 654 (34.3%) and 764 (39.5%), respectively. •Versus 147 (28.9%) non-Hispanic White patients	Mortality and ICU admissions	•Rates of death: 20.0% non-Hispanic White patients (102 of 509), 17.2% non-Hispanic Black patients (333 of 1935), 16.2% Hispanic patients (309 of 1905), 17% Asian patients (29 of 171) •470 patients needed a stay in the ICU
**9**	Yehia, B. R. (2020) [21]	USA	Cohort Study	11210	•Black •White •Other	•Asthma •Chronic Kidney Disease •Chronic Liver Disease •Chronic Obstructive Pulmonary Disease •Congestive Heart Failure •Coronary Artery Disease •Diabetes •Hypertension •Obesity	•In comparison to White patients, a greater rate of Black patients had asthma (216 [4.7%] vs 367 [8.8%]), cancer (145 [3.2%] vs 151 [3.6%]), chronic kidney disease (595 [12.9%] vs 858 [20.5%]), congestive heart failure (496 [10.8%] vs 521 [12.5%]), diabetes (1061 [23.0%] vs 1337 [32.0%]), hypertension (1153 [25.0%] vs 1265	ICU admissions and Mortality	•Mortality for White patients was 23.1% (724 of 3128). •Mortality for Black patients was 19.2%)540 of 2812). •36.4% of White patients that experienced an ICU stay died (477 of 35.2%). •35.2% of Black patients that experienced an ICU stay died (388 of 1102).

						•Solid Organ Transplantation	[30.3%]), obesity (838 [18.2%] vs 1345 [32.2%]), and solid organ transplantation (14 [0.3%] vs 21 [0.5%]) •Hospitalized Black patients presented with more comorbidities than hospitalized White patients		
**10**	Best, J. H. (2020) [22]	USA	Retrospective, observational study	3471	•African American •White	•Hyperlipidemia •Pulmonary Fibrosis •Chronic Obstructive Pulmonary Disease •Coronary Artery Disease •Diabetes •Hypertension •Obesity •Asthma •Sleep Apnea	•Higher rates of diabetes (19.8% vs. 16.7%; P = 0.032) and obesity (11.6% vs. 9.0%; P = 0.025) and a higher mean (SD) BMI (33.66 [9.46] vs. 30.42 [7.86]; P < 0.001) and median BMI (32.20 vs. 29.52) shown in African American patients compared to White patients •White patients had greater rates of hyperlipidemia (19.6% vs. 16.5%; P = 0.030), chronic obstructive pulmonary disease (COPD; 8.2% vs. 5.6%; P = 0.005), and self-reported a history of smoking/tobacco use (37.2% vs. 28.1%; P < 0.001) in comparison to African American patients.	NA	NA

**11**	Alkhatib, A. L. (2020) [23]	USA	retrospective cross-sectional analysi	158	•African American	•Obstructive Lung Disease (Asthma & Chronic Obstructive Pulmonary Disease)<br>•Chronic Kidney Disease (CKD)<br>•Congestive Heart Failure<br>•Diabetes Mellitus (DM)<br>•Hypertension	•Findings: in African American patients, age, greater BMI and obstructive lung disease were associated with severe COVID-19	ICU admissions and Mortality	•85% of patients with severe cases of COVID-19 that were admitted to the ICU has respiratory failure and they needed intubation and mechanical ventilation<br>•ICU mortality was 37% for severe cases
**12**	Yu, Y. (2021) [40]	USA (Michigan)	Retrospective cohort study	249075	•Black<br>•White	• Respiratory conditions<br>• Circulatory conditions<br>• Cancer<br>• Type 2 diabetes<br>• Kidney diseases<br>• Liver diseases<br>• Autoimmune diseases	All the comorbidity conditions considered were associated with an increased risk of testing positive. In the full cohort, Black patients had significantly higher risk of testing positive than White patients. The main effects of the seven comorbid conditions considered were significant in both Black and White patients for hospitalisation, while none of the comorbidities exhibited significant interaction effects	ICU admissions and Mortality	Black patients had significantly higher (compared to White patients) hospitalisation rates (19.6% *vs.* 11.0%) and ICU admission rates (6.3% *vs.* 2.8%) in the full COVID-19-positive cohort. 844 (3.2%) were admitted to the ICU, and 485 (1.8%) died
**13**	Dai, C. L. (2021) [59]	USA (California, Oregon, and Washington)	Retrospective cohort study	629953	• Non-Hispanic White<br>• Hispanic<br>• Non-Hispanic Asian American<br>• Non-Hispanic	• Charlson Comorbidity Index (CCI)<br>• Obesity<br>• Hypertension	• The most common comorbidities were obesity (37.1%), hypertension (23.3%), diabetes (9.4%), and asthma (6.5%)<br>• The prevalence of obesity was highest among American Indian/Alaska Native,	Mortality	• Hispanic race/ethnicity was significantly associated with increased odds of in-hospital mortality

					Black • Non-Hispanic American Indian/Alaska Native • Non-Hispanic Native Hawaiian/Pacific Islander • Non-Hispanic other • Unknown		Native Hawaiian/Pacific Islander, and Hispanic patients and lowest among Asian patients • Hispanics had the lowest prevalence of hypertension, whereas Asian and Black patients had the highest		
**14**	Kalyanaraman M. R. (2020) [46]	USA (New York)	Retrospective study	22254	• Black • White • Hispanic	• Diabetes • Hypertension (HTN) • Arrhythmia • Cardiovascular disease (CVD) • Congestive heart failure (CHF) • Asthma • Chronic obstructive pulmonary disease (COPD) • Chronic kidney disease (CKD) • Liver disease • Cancer	• Hispanic and Black patients had a higher prevalence of obesity than other groups, with 13% of Blacks having a BMI ≥40 compared to 6% of Hispanics and 8% of Whites • Black patients were also more likely to have one or more chronic diseases than other racial/ethnic groups (64% of Black patients vs. 50% of Hispanic patients and 52% of White patients) • Black patients had a higher prevalence of diabetes, hypertension, cardiovascular disease (CVD), congestive heart failure (CHF), and chronic kidney disease (CKD) • White patients who died had the highest prevalence of	Mortality	• Blacks and Hispanics each comprised approximately one-third of all patients who died (29% and 31%, respectively), and Whites comprised 12% • White race was significantly associated with death, with a greater proportion of Whites dying than among all other racial/ethnic groups (47% for Whites vs. 32% for Blacks, 31% for Hispanics)

							arrhythmia (17%), CVD (36%), and CHF (15%), and chronic obstructive pulmonary disease (COPD) (7%) of all racial/ethnic groups		
**15**	Izzy, S. (2020) [60]	USA (Massachusetts)	Retrospective study	5190	• White<br>• Latinx<br>• African American<br>• Asian American	• Obesity<br>• Diabetes mellitus<br>• Hyperlipidemia<br>• Hypertension<br>• Coronary heart disease<br>• Congestive heart failure<br>• Chronic obstructive pulmonary disease<br>• Asthma<br>• Interstitial lung disease<br>• Cerebrovascular disease<br>• Chronic kidney disease<br>• End-stage renal disease<br>• Malignancy including hematologic malignancy	• Hospitalized Latinx patients were more likely to be obese in the range of 30–40 kg/m2 (41% compared with 31% among White and 33% among African American patients; P < .001)<br>• The proportions of White, Latinx, and African American patients who were in the range of >40 kg/m2 were similar<br>• White patients had higher rates of reported hyperlipidemia, hypertension, obstructive lung disease, coronary artery disease, congestive heart failure, chronic kidney disease, and malignancy compared with other groups (P < .05)<br>• Latinx and African	ICU admissions	• Latinx and African American patients disproportionally required admission to the ICU compared with White patients<br>• 39% of Latinx patients required admission to the ICU compared with 33% of African American patients, 24% of Asian American patients, and 30% of White patients

						(lymphoma, leukemia)<br>• HIV<br>• History of organ or bone marrow transplantation	American patients were admitted at twice the rate of Whites, regardless of reported pre-existing comorbidities (P < .001)		
**16**	Gu, T. (2020) [41]	USA (Michigan)	Retrospective cohort Study	5698	• Non-hispanic White<br>• Non-hispanic Black	• Respiratory conditions<br>• Circulatory conditions<br>• Cancer<br>• Type 2 diabetes<br>• Kidney disease<br>• Liver disease<br>• Autoimmune disease	• Higher risk of hospitalization was observed in patients with pre-existing type 2 diabetes (OR, 1.82 [95% CI, 1.25-2.64]; P = .02) and kidney disease (OR, 2.87 [95% CI, 1.87-4.42]; P < .001)<br>• Compared with White patients, obesity was associated with higher risk of having test results positive for COVID-19 among Black patients<br>• Overall comorbidity burden was associated with higher risk of hospitalization in White patients (OR, 1.30 [95% CI, 1.11-1.53]; P = .001) but not in Black patients (OR, 0.99 [95% CI, 0.83-1.17]; P = .88; P for interaction = .02), as was type 2 diabetes	Mortality and ICU admissions	• No statistically significant racial differences were found in ICU admission and mortality based on adjusted analysis

**17**	Parker, J. J. (2021) [61]	USA (Tennessee)	Retrospective study		• White, non-Hispanic • Black, non-Hispanic • Hispanic, regardless of race	• Cardiovascular disease • Chronic liver disease • Hypertension • Chronic kidney disease • Diabetes	• Hispanic patients had the lowest prevalence of underlying medical conditions (64.3%) compared with White patients (85.7%) and Black patients (91.3%) • Hypertension, CVD, chronic liver disease, cancer, chronic renal disease, diabetes, and a history of smoking were more common among deceased case-patients • Surveillance group: Hispanic patients had a lower number of comorbidities than White and Black patients • Chart-abstracted group: number of comorbidities for Hispanic patients was twice that of the standard surveillance group, which was the largest increase for race/ethnicity	Mortality	• Deaths of COVID-19 case-patients were more likely to be in older, male, and Black case-patients • There was a difference in the case-fatality rate (CFR) for COVID-19 by race and ethnicity (White 1.9%, Black 2.6%, and Hispanic 0.5%) • 29.8% of deaths were in Black persons • Hispanic patients accounted for 7.0% of deaths • White patients represented 60.3% of the deaths but 73.5% of the population
**18**	Lobelo, F. (2021) [53]	USA (Atlanta)	Retrospective cohort study	5712	• Black • White • Hispanic • Asian • Other	• Hypertension • Diabetes • Obesity • Hyperlipidemia • CAD • Congestive heart failure • Asthma • COPD • Arrhythmia • HIV • Depression • Cancer	• Black patients had the highest rates of obesity (9%), hypertension (34.7%), asthma (11.3%) and HIV (1.9%) • White patients had the highest rates of congestive heart failure (7.2%), coronary artery disease (7.4%), arrhythmia (5%), chronic obstructive pulmonary disease (4.6%), depression (15.5%)	ICU Admissions	• Asians had the highest rates of ICU admission (53.1%) and mechanical ventilation (21.9%)

							• Asian patients had the highest rate of diabetes (18%)		
**19**	Marmarchi, F. (2021) [54]	USA (Atlanta)	Observational cohort Study	288	• African American	• Hypertension<br>• Coronary artery disease<br>• Asthma<br>• Diabetes	• African Americans had significantly higher rates of comorbidities compared to other races, including hypertension (80% vs 59%, P = 0.001), diabetes (49% vs 34%, P = 0.026), and mean BMI (33 kg/m2 vs 28 kg/m2, P < 0.001)<br>• No differences between races in terms of coronary artery disease, stroke, chronic kidney disease, chronic obstructive pulmonary disease, asthma, and sleep apnea	ICU admissions and Mortality	• No significant difference in deaths between African Americans and non-African Americans

**20**	Kolinski, J. M. (2020) [62]	USA (Wisconsin)	Retrospective analysis	168	• African American • White • Other	• Chronic kidney disease and/or dialysis • Dementia • Diabetes mellitus • Heart failure • Hypertension • Obesity • Asthma • Cancer • COPD • CAD	• African American patients were more likely to have chronic kidney disease or dialysis dependency, hypertension, diabetes, and higher levels of C-reactive protein and procalcitonin	ICU admissions	• 89 patients (53%) spent time in ICU • 31 patients (18%) underwent mechanical ventilation • Patients who spent time in the ICU were more likely to have diabetes and/or obesity
**21**	Casillas, E. (2021) [55]	USA (California)	Retrospective cross-sectional study	278	• Hispanic • Non-Hispanic	• Diabetes • Hypertension	• Among Hispanic patients with pre-existing conditions, diabetes was more prevalent while hypertension was less prevalent in comparison to non-Hispanic patients	ICU admissions and Mortality	• Transfer to ICU rate, ICU mortality, and overall mortality did not significantly differ between Hispanic and non-Hispanic patients
**22**	Musshafen, L. A. (2022) [64]	USA (Mississippi)	Retrospective cohort study	1230	• American Indian/Mississippi Band Choctaw Indian • African American/Black • Caucasian/White • Asian or Native Hawaiian/Pacific Islander • Other	• Cancer • Chronic kidney disease • COPD • Down syndrome • Heart failure • Coronary artery disease • Cardiomyopathies • Obesity • Severe obesity • Sickle cell disease • Type 2 diabetes mellitus	• Comorbidities most frequently observed among American Indians were type 2 diabetes (67%, n=41), obesity (41%, n=25), chronic kidney disease (25%, n=15), and smoking (25%, n=15) • Type 2 diabetes was the condition most observed among American Indians who died while in hospital, with 72% (n=18) of the 25 American Indians who died having the condition • Chronic kidney disease (28%, n=7), obesity (28%, n= 7), and heart conditions (24%, n=6) were also	Mortality	• While the mortality prevalence for all adults admitted with a primary diagnosis of COVID-19 was 20.8% (n=256) in unadjusted statistics, the mortality prevalence among American Indians was more than double at 41% (n=25) • 22.6% of Caucasians/Whites (n=65), 18.9% of African Americans/Blacks (n=153), and 18.6% of inpatients with an Other/Unknown race (n=13) died while in hospital • The adjusted probability of inpatient mortality among American Indians was 46%, which was greater than that of all other races

							commonly observed among those who died before discharge		
**23**	Lopez, D. C. (2022) [65]	USA (Cleveland)	Retrospective cohort study	2125	• Black<br>• White<br>• Other (Asian, Native Hawaiian, Pacific Islander, American Indian, Alaskan Native, multiracial, non-Hispanic, and Hispanic)	• Diabetes<br>• Asthma<br>• CKD<br>• chronic cardiac disease<br>• COPD<br>• CAD<br>• Dementia<br>• Hypertension<br>• Hematological malignancies<br>• Solid organ and bone marrow transplants<br>• Malnutrition<br>• Liver disease<br>• Immunodeficiencies	• Black patients had a significantly higher prevalence of asthma (26.3% vs. 20.4%, p = 0.003), CKD (48.6% vs. 36.7%, p < 0.001), and diabetes (68.1% vs. 56.0%, p < 0.001) as compared to White patients<br>• Black patients had a lower prevalence of malignant neoplasms (37.3 vs. 45.0%, p = 0.001)<br>• There were no significant differences between Black and White patients for chronic cardiac disease, COPD, CAD, dementia, hypertension, hematological malignancies, solid organ and bone marrow transplants, malnutrition, liver disease, and immunodeficiencies<br>• As compared to non-Hispanic patients,	ICU admissions and Mortality	• Race/ethnicity were not found to be associated with mortality

							Hispanic patients within this cohort had a significantly lower prevalence of chronic cardiac disease (52.8% vs. 71.2%, p < 0.001), CKD (20.2% vs. 41.5%, p < 0.001), COPD (23.6% vs. 36.3%, p = 0.015), hypertension (80.9% vs. 89.9%, p = 0.007), and malignant neoplasms (29.2% vs. 41.8%, p = 0.019) • Hispanic patients had a significantly higher prevalence of liver disease (32.6% vs. 22.2%, p = 0.022) • No significant differences were observed in the prevalence of asthma, CAD, diabetes, solid organ and bone marrow transplant, malnutrition, HIV, and immunodeficiency disorders between groups		
**24**	Zakaria, A. (2021) [42]	USA (Michigan)	Retrospective observational study	1064	• African American • Non-Hispanic White	• Chronic kidney disease • Dementia • Chronic kidney disease • Body Mass Index	•The Charlson Comorbidity Index score was the same in African American and non-Hispanic White populations •African Americans were more likely to have chronic kidney disease (CKD) compared to non-Hispanic White patients •Non-Hispanic White	Mortality	• All-cause in-hospital mortality was 21.7% • Patients with a higher Charlson Comorbidity Index score, dementia, CKD, and lower BMI were all associated with higher mortality

							patients were more likely to have dementia		
**25**	Shaw, P. A. (2022) [66]	USA (Pennsylvania)	Retrospective study	6953	•Black<br>•White<br>•Hispanic/Latinx	•Diabetes (Type 1 or Type 2)<br>•Obesity<br>•Chronic kidney disease<br>•Chronic liver disease,<br>•Chronic respiratory disease (COPD, asthma, and chronic oxygen requirement)<br>•Cardiovascular disease (coronary artery disease, hypertension, congestive heart failure, and other)<br>•Immune deficiency	•Black patients were more likely to have at least one comorbid condition (71.3% versus 56.6% in Whites and 48.2% other races)<br>•Diabetes, kidney disease, respiratory disease, and cardiovascular disease were more common in Blacks than all other races<br>•Hispanic/Latinx patients were less likely to have one or more comorbid conditions compared to non-Hispanic/Latinx (42.8% vs 64.2%)	Mortality and ICU Admissions	•Black patients were more likely to require ICU admission compared to all other races (15.2% versus 13.6% for Whites and 18.8% for other races)

**26**	Kodsup, P. (2022) [67]	USA (Louisiana)	Retrospective observational study	408,047	•African American •White American	•Hypertension (59.4%) •Diabetes (36.7%) •Cardiac disease (21%) •Kidney disease (20%) •Obesity (20%) •Heart failure (13%) •Chronic obstructive pulmonary disease (COPD) (11.7%) •Neurological (8.43%) •Cancer (7.51%) •Asthma (4.15%)	•The mortality rate among AA was positively associated with diabetes, hypertension, and overweight BMI	Mortality	•AA COVID-19 death was three times higher than other races •Mortality rate was 10 times higher in counties with more than 40% AA
**27**	Abate, G. (2022) [69]	USA (Midwest)	Retrospective study	N/A	•African Americans •Caucasians	•Chronic kidney disease •COPD •Diabetes •Congestive heart failure	•COPD was more common in Caucasian patients •In African American patients who died, a history of COPD, diabetes mellitus, and congestive heart failure were significant	Mortality and ICU Admissions	•There was no significant difference in hospital mortality between African Americans and Caucasians(20.9% AA, 21.5% Caucasian and 12.5% other race •ICU stay did not differ among the different races •African Americans have higher rates of COVID-19 associated mortality
**28**	Shadyab, A. H. (2021) [56]	USA (California)	Retrospective cohort study	100	•Hispanic (46%) •Non-Hispanic (4.0% White, 10.0% Black, 6.0% Asian, 39.0% other race or mixed race)	•Hypertension •Coronary artery disease •Chronic liver disease •Diabetes	•Obesity was significantly higher in Hispanic relative to non-Hispanic patients with COVID-19 •The percentage of comorbidities was not significantly different between Hispanic compared with non-Hispanic patients with COVID-19	Mortality and ICU Admissions	•79.0% of patients with COVID-19 were hospitalized •19.0% of patients with COVID-19 died •The rate of hospitalization or death was not significantly different between Hispanic and non-Hispanic patients with COVID-1. •The rates of other poor outcomes (ICU admissions) were not significantly different

									between Hispanic and non-Hispanic patients with COVID-19
**29**	Cervantes, J. (2021) [70]	USA (Mexico border)	Retrospective analysis	82	•Hispanic	•Coronary artery disease •Cerebrovascular disease •Cancer •Chronic kidney disease •Asthma •Cirrhosis •Hepatitis •Hypothyroidism •HIV •Inflammatory bowel disease	•The most common comorbidities were hypertension (48.8%) and diabetes (39%)	Mortality, ICU admissions	•A total of 15 in-hospital deaths (18.3%; 95% CI) and 24.4% ICU admissions (n=20)
**30**	Ricardo, A. C. (2022) [24]	USA	Cohort study	2153	•Hispanic (46.2%) •Non-Hispanic White (53.8%)	•Hypertension •Chronic obstructive pulmonary disease •Coronary artery disease •Heart failure	•Hispanic patients were less likely to have hypertension, chronic obstructive pulmonary disease, coronary artery disease, or heart failure	ICU admissions and Mortality	•785 patients (36.5%) died •Hispanic patients had higher odds of death compared with non-Hispanic White patients
**31**	Price-Haywood, E. G. (2020) [68]	USA (Louisiana)	retrospective cohort study	3481	•Black non-Hispanic (70.4%) •White non-Hispanic (29.6%)	•Obesity •Diabetes •Hypertension •Chronic kidney disease	•Black patients had higher prevalences of obesity, diabetes, hypertension, and chronic kidney disease than White patients	ICU admissions and Mortality	•Of 326 patients who died from COVID-19, 70.6% were black •There was no association between in-hospital mortality and race
**32**	Xie, J. [71]	USA (New Orleans)	retrospective, observational study	287	•Black/African American	•Acute respiratory distress syndrome •Hypertension •Obesity •Diabetes	•Hospitalized Black patients with the clustering of hypertension, obesity, and diabetes increased the odds of mortality compared with these	ICU admissions	•Obesity in non-Hispanic Black patients was associated with increased odds of ICU requirement •MetS (metabolic syndrome) in non-Hispanic black patients was

						•Metabolic syndrome	comorbidities individually		also associated with hospital mortality
**33**	Page-Wilson, G. (2021) [47]	USA (New York)	retrospective cohort study	928	•Black •Asian	•Obesity	•Black and Asian patients were more likely to require renal replacement therapy	Mortality	•Severe complications of COVID-19 and death are more likely in patients with obesity, independent of age and comorbidities
**34**	Raiker, R. (2021) [25]	USA	Cohort study	9730 rheumatoid arthritis 656,979 non rheumatoid arthritis	•White •Black •Asian	•Rheumatoid arthritis (RA) •Hypertension •Chronic lower lung disease •Diabetes mellitus •Ischemic heart disease •Chronic kidney disease •Heart failure •Cerebrovascular disease		Mortality and ICU admissions	•The risk of all outcomes was higher in the RA compared to the non-RA cohort before matching •No difference in the majority of outcomes after matching •The risk of mortality, hospitalization , ICU admission, mechanical ventilation , severe COVID-19, acute kidney injury, ARDS, ischemic stroke, and sepsis was higher in black compared to the white race •The risk of KRT/hemodialysis was not statistically significantly different
**35**	Nguyen, J. L. (2022) [26]	USA	retrospective cohort study	679 566	•Black/African American	•Asthma •Cardiovascular disease •Cerebrovascular disease •Chronic kidney disease •Chronic liver disease •COPD •Diabetes •Hypertension •Malignancy (including melanoma but no other skin	•Black patients had the highest rates of cardiovascular disease and hypertension across all age groups compared to other races	ICU admissions and Mortality	•PAFs were generally higher in Black persons than in other race/ethnicity groups for the same conditions, particularly in the 2 younger age groups. •Black and Hispanic patients were more likley to be hospitalized and die from COVID-19

						cancers)<br>•Neurologic disease<br>•Obesity<br>•Rheumatic disease			
**36**	Arasteh, K. (2020) [27]	USA	Retrospective study	10 076	•Black<br>•Hispanic<br>•White	• Hypertension<br>• Diabetes	• Hypertension and diabetes prevalence was greater among Blacks and Hispanics compared with whites among all age groups<br>• Among the cohort over 65 years old, 74% of Blacks and 69% of Hispanics reported hypertension, compared with 50% of Whites<br>• Among those over 65 years old, 30% of Blacks and 39% of Hispanics reported diabetes, compared with 20% of Whites<br>• Among those over 65 years old, 79% of Black individuals and 78% of Hispanic individuals reported either hypertension or diabetes compared to 56% of White individuals	Mortality	• Greater rates of comorbidities associated with COVID-19 among Blacks and Hispanics put them at a greater risk of death from COVID-19 infection<br>• As the older population is more vulnerable to COVID-19, the greater prevalence of comorbidities among Blacks and Hispanics 65 years of age and older puts these populations at greater risk

**37**	Racine , R. (2022) [72]	USA (Southwest Georgia)	Retrospective Cohort Analysis	710	• African American • Non African American (Caucasian, Asian, Other Race)	• Hypertension • Coronary Artery disease • Congestive hart failure • COPD • Asthma • Chronic kidney disease • Diabetes • Cancer • Chronic liver disease • Dementia • Stroke • Obesity	• African americans were more likely to have hypertension as a baseline comorbidity • Non-African Americans were more likely to have coronary artery disease (CAD), chronic obstructive pulmonary disease (COPD), and chronic liver disease.	Mortality	• African Americans have a lower risk of in hospital mortality than non-African American (16.1% vs 17.8%, p = 0.9) • African Americans also had a lower risk of ICU admission compared to non-African Americans (25.8% vs 30.5%, p = 0.71) • Both of these observations were NOT statistically significant
**38**	Wiley, Z. (2022) [28]	USA	Cohort study	94 683	• Hispanic • Black • White	• Hypertension •Diabetes • Obesity • Morbid obesity • Congestive heart failure • Coronary artery disease • Chronic kidney disease • End stage renal disease • Liver disease • Asthma • Chronic obstructive pulmonary disease •Obstsructive sleep apnea • cancer		Mortality	• Both Hispanics and Black patients had overall increased RRs of in hospital mortality compared to white patients (RR = 1.28 [95% CI: 1.13-1.44;p <.001] and RR = 1.18 [95% CI: 1.06 - 1.31; P = .002], respectively)

**39**	Rodriguez, F. (2020) [29]	USA	Retrospective observational study	8590	• NH White<br>• NH Black<br>• Hispanic (All races),<br>•Asian/Pacific Islander	• Obesity<br>• Diabetes<br>• Hypertension<br>•Coronary artery disease<br>• Heart failure<br>•cerebrovascular disease<br>•peripheral artery disease<br>•atrial fibrillation/flutter<br>•pulmonary disease<br>•pulmonary embolus<br>•deep venous thrombosis<br>•immune disorder	•Half of hospitalized Black patients were obese at 49.3% compared with just 17.7% of hospitalized Asian patients.<br>•Black patients had the highest prevalence of obesity, hypertension, diabetes, prior cerebrovascular disease, and advanced kidney disease on admission.<br>•NH White patients had the highest prevalence of prior coronary artery disease and pulmonary disease in commparison with other racial/ethnic groups.	Mortality	• 53.1% of all mortality occurred in Black or Hispanic patients<br>•In hospital mortality was observed in 17.6% of Black, 16.0% of Hispanic, and 19.3% of Asian patients compared with 21.1% of non-Hispanic White patients.<br>•Adjusted odds ratios (ORs) for mortality were 0.93 (95% CI, 0.76-1.14) for Black patients, 0.90 (95% CI, 0.73-1.11) for Hispanic patients, and 1.31 (95% CI, 0.96-1.80) for Asian patients compared with NH White patients.
**40**	Elbadawi, A. (2022) [73]	USA (Northeast Ohio, South Florida)	Observational cohort Study	3678	•Caucasian<br>•Black<br>•Hispanic<br>•Other	•Hypertension<br>•diabetes<br>•heart failure<br>•coronary artery disease<br>•chronic<br>•obstructive pumonary disease<br>•bronchial asthma	1 - Caucasian patients were more likely to have a history of chronic obstructive pulmonary disease (COPD), coronary artery disease (CAD), heart failure, cancer, and connective tissue disease.<br>2 -Black patients were more likely to have hypertension and diabetes.	Mortality and ICU admission	1 - In-hospital mortality was lower in Black patients compared to Caucasian patients. No difference observed with other racial groups<br>2 - The frequency of critical illness (ICU admission, mechanical ventialtion, ore pressor support) was lower among Hispanic patients while no difference was observed with Black patients

**41**	Metra, B. (2021) [30]	USA	Retrospective observational cohort study	346953	•non-Hispanic Black<br>•non-Hispanic White	•Hypertension<br>•diabetes<br>•obesity<br>•chronic obstructive pulmonary disease<br>•cerebral infarction<br>•systemic connective tissue disorders,<br>•reduced mobility<br>•pregnancy<br>•neoplasm	1 - Black COVID-19 patients had higher prevalence of diabetes, hypertension, and obesity<br>2 - White patients had.a higher prevalence of neoplasms and COPD	Mortality and ICU admission	1 - Within matched cohorts (Blacks with pulmonary embolism) Blacks with COVID-19 had a higher mortality rate at 30 days (RR 1.890, p < 0.0001), as well as higher rates of hospitalization, ICU care and mechanical ventilation within 30 days of COVID-19 diagnosis
**42**	Krishnamoorthy, G. (2020) [43]	USA (Michigan)	Retrospective observational study	799	•Black<br>•White	•Congestive heart failure<br>•diabetes<br>•chronic obstructive pulmonary disease	•No association between the presence of diabetes in those under the age of 65 compared with those 65 and above in White patients (27% vs. 34.3%, P ¼ .2)<br>•Black patients under 65 had a lower rate of diabetes than Black patients 65 and above (34.9% vs. 46.9%, P ¼ .03)	Mortality	1 - White patients with diabetes had a higher mortality rate (43.8%) as compared to Black patients with diabetes

**43**	Parpia, A. S. (2021) [44]	USA (Michigan)	Retrospective study	6065	•White •Black •Native American •South Asian •Southeast Asian •East Asian •other Asian •Hispanic/Latinx •non-Hispanic/Latinx •other/not reported	•Asthma or reactive airway disease •cardiovascular disease •cancer •chronic lung disease •diabetes mellitus •neurologic disease •chronic liver disease •chronic renal disease •other immunosuppressive conditions •other chronic diseases	•The most common comorbidity dyads among all COVID-19 deaths were diabetes and cardiovascular disease (24.4%), and cardiovascular and neurologic disease (21.6%). •Black patients had significantly higher rates of reporting asthma (p = 0.016), diabetes (p = 0.002), and chronic renal disease (p = 0.004) than White individuals who died from COVID-19, but significantly lower rates of cardiovascular disease (p<0.001), cancer (p<0.001), chronic lung disease (p<0.001), and neurologic disease (p<0.001). •Black patients had higher rates of to have the combination of diabetes mellitus, cardiovascular disease, and chronic renal disease (p = 0.003) than White patients, and lower rates of the combinations of cardiovascular and chronic lung disease (p<0.001) and cardiovascular and neurologic disease (p<0.001)	Mortality	•Black males aged 65 and older with multiple comorbidities had a mortality rate that was 3.5 times that of White males with multiple comorbidities

**44**	Escobar, G. J. (2021) [57]	USA (Northern California)	Retrospective cohort study	3481716	•White •African American •Hispanic •Asian •Other/unknown	•Hypertension •diabetes •obesity •chronic obstructive pulmonary disease •congestive heart failure •cancer	•Compared with the other groups, Black/African Americans had higher rates of diabetes, obesity, hypertension, chronic pulmonary disease, and congestive heart failure	Mortality	1 - Unadjusted mortality rates were highest for White persons (17.0% during their first hospitalization and 27.7% during thestudy period), followed by Black/African American persons (12.7% and 19.0%, respectively), Asian persons (10.5% and 14.8%), persons of other/unknown race (8.1% and 11.3%), and Hispanic persons (9.7% and 10.3%). 2 - Adjusted analyses showed no racial differences in inpatient mortality or total mortality during the study period
**45**	Muñoz-Price, L. S. (2020) [63]	USA (Wisconsin)	Cross-sectional study	369	•Black •White •Native Hawaiian •Native American or Alaska Native •Asian •Unknown	•Hypertension •Diabetes •Chronic heart disease •chronic lung disease •chronic kidney disease	•Proportion of African American patients with 3 or more comorbidities was higher than that of patients who belonged to other racial groups (28.9% vs 22.4%, p = 0.001) as was the mean BMI (33.2 vs 30.8, p = 0.001)	(In-hospital) Mortality and ICU admissions	•17.2% overall in-hospital mortality rate (17.9% for African Americans, 15.9% for other racial groups) •19% overall ICU admission rate (20.2% for African Americans, 17.2% for other racial groups)
**46**	Egede, L. E. (2020) [74]	USA (Milwaukee and Southeast Wisconsin)	cross-sectional analysis	31,549	•4.5 percent of non-Hispanic Whites •14.9 percent of non-Hispanic Blacks •14.8 percent of Hispanics	•Congestive heart failure •Cardiac arrhythmias •Hypertension •Chronic pulmonary disease •Diabetes •Renal failure •Cancer •Fluid •Electrolyte disorders •Anemia •Depression	• After adjustment for demographics and comorbidities, Black patients and Hispanic patients were more than 3x more likely to screen positive and 2x more likely to be hospitalized compared to White patients •Hispanics were 2x more likely to experience mortality than Whites	Mortality and ICU admissions	• Unadjusted proportions for an ICU stay during hospitalization by race were 10.0% overall (95% CI, 8.8, 11.3) and 7.8% for non-Hispanic White (95% CI, 6.2, 9.4), 12.5% for non-Hispanic Black (95% CI, 10.4, 14.6), and 10.3% for Hispanic (95% CI, 6.3, 14.2) adults (p = 0.002) •Unadjusted proportions for mortality by race were 5.4% overall (95% CI, 4.5, 6.4) and 4.5% for non-Hispanic White (95 percent CI 3.3, 5.7), 6.3% for non-Hispanic Black (95% CI, 4.7, 7.8), and 6.3% for Hispanic (95% CI, 3.1, 9.4) adults

**47**	Bushman, D. (2021) [48]	USA (New York)	Case-control study	15 097	•NH Asian •NH Black •Hispanic/Latino •NH White •NH Other	•Heart disease •congestive heart failure •coronary artery disease •hyperlipidemia •valvular heart disease •obesity •diabetes •lung disease •asthma •chronic obstructive pulmonary disease •kidney disease •renal failure on dialysis •anemia •sleep apnea •peripheral vascular disease •liver disease •viral hepatitis •hepatitis C virus infection •autoimmune disease •gastroesophageal reflux disorder •history of stroke •active tuberculosis	•Five of these conditions were most prevalent among Black control patients, including heart disease (74.9%), obesity (61.5%), diabetes (43.8%), lung disease (24.6%), and kidney disease (13.4%) compared to patients of other races/ethnicities.	Mortality	•Patients with both heart disease and diabetes had 3.13 times the odds of death compared to patients who had neither condition •Patients with heart disease had 1.97 odds of death while patients with diabetes had 2.57 times.

**48**	Shakil, S. S. (2022) [31]	USA	Retrospective Cohort Study	21 073	•Hispanic •NH black •NH White •Asian •Other	•Ischemic stroke and TIA (Transient ischemic attack) •Other stroke •Obesity •Diabetes •Hypertension •Coronary artery disease •Heart failure •Cerebrovascular disease •Atrial fibrillation/flutter •Chronic kidney disease	•NH Black patients accounted for 30.6% of ischemic stroke/TIA while being 25.7% of the registry. •Asian patients acounted for 6.9% of ischemic stroke/TIA while being 3.9% of the registry. •Hispanic patients accounted for 17.5% of ischemic stroke/TIA while being 25.6% of the registry. • NH White accounted for 38% of ischemic stroke/TIA while being 37.5% of the registry. •Acute ischemic stroke prevalence was 1.3% among Asian patients, 0.91% among NH Black patients, 0.75% among NH White patients and 0.52% among Hispanic patients. •Odds of ischemic stroke/TIA did not differ after sequential adjustment for dmeographics, comorbidities and illness severity.	Mortality	• In hospital mortality was higher among stroke patients than no stroke patients (37.5% for stroke and 15.1% for no stsroke).
**49**	Toth, A. T. (2021) [49]	USA (New York)	Retrospective Cohort Study	2233	•Hispanic •non-Hispanic Black/African American •NH White •NH Asian/Pacific islander •NH other	•Diabetes •Hypertension •asthma •heart disease •congestive heart failure •chronic kidney disease	N/A	Mortality while in ICU stay	In hospital mortality rate in Hispanic patients was significantly higher during periods of high/very high surge compared with low/medium surge (69.9% vs 56.4%). Other groups not found to be significantly different.

**50**	Ashktorab, H. (2022) [32]	USA	Retrospective study	5852	•African American •European American •Latin American •Asian American	•Cardiac disease •Diabetes •Hypertension •Obesity	•Cardiac disease, African Americans (13.8%), European Americans (13.1%), Latin Americans (4.5%), Asian Americans (4%). •Asthma, African Americans (11.1%), EA (7.7%), LA (6.9%), Asian Americans (5.7%). •Diabetes mellitus, African American (39%), EA (24.6%), LA (22.8%), Asian American (22.6%). •Hypertension, African American (65%), EA (45.8%), LA (30.7%), Asian American (34.4%). •Overall African Americans had the highest prevalence of comorbidities over the other races.	Mortality	•EA had the highest mortality rate of 17% vs. 14.8% for African Americans and 7.3% for Latin Americans. •After adjusting for comorbidities, African Americans had the highest risk of Covid19 death.
**51**	Ebinger, J. E. (2021) [58]	USA (California)	Retrospective Cohort Study,	1584	•Asian •Hispanic/Latinx •NH Black •NH White	•Hypertension •Diabetes •Obesity •Priod COPD or asthma •Prior myocardial infarction or heart failure	•Obesity, NH White (18.9%), Asian (6.5%), Hispanic/Latinx (20%), NH Black (24.8%). •Hypertension, NH White (50.5%), Asian (45.2%), Hispanic/Latinx (36.5%), NH Black (59.9%). •DIabetes, NH White (26%), Asian (35.5%), Hispanic/Latinx (32.4%), NH Black (31.3%).	ICU Admission	•Hispanic/Latinx patients were more than twice as likely to require ICU admission than NH White patients. •Hispanic/Latinx patients were morel ikely to experience severe illness compared with NH Whites.
**52**	Golestaneh, L. (2020) [50]	USA (New York)	Retrospective Cohort Study	505 992	•NH White •NH Black •Hispanic •Other	•Diabetes •Asthma •Hypertension •Obesity	•Black patients compared to white patients, diabetes (47.2% vs 25.8%) and asthma (21.9% vs 15.8%) •Black patients suffer from more diabetes and	Mortality	•Black patients had no increase in in hospital mortality compared to white patients. •The parent population of Black patients suffered disproportionate higher mortality than the White patients.

							obesity compared to whites.		
**53**	Samuels, S. (2021) [75]	USA (Florida)	retrospective analysis	1692	•NH Black •NH White •Hispanic	•Hypertension •Obesity •Heart disease •Pulmonary disease •Diabetes •Asthma •Kidney disease •Rheymatologic disease •Liver disease •Hypothyroidism •Dementia •Neurological disorder •Hematologic disease	•NH white patients had 3 or more underlying conditions (35.8%) compared to NH Black (26.6%) and Hispanic (21.6%)	Mortality and ICU admission	•Case fatality rate, NH White 14%, Hispanics 4.1%, NH Black 3.3%. ICU admissions, NH White (22.4%), NH Black (32.8%), Hispanic (44.8%).
**54**	Navar, A. M. (2021) [33]	USA	Retrospective cohort study	19 584	•Black or African American •Hispanic •White •American Indian or Alaska •Asian or Pacific Islander •Mixed racial group	•Hypertension •DIabetes •Asthma •Coronary Artery disease •Chronic obstructive pulmonary disease •end stage renal failure	•Black patients had higher rates of diabetes, hypertension, coronary heart disease, heart failure, chronic kidney disease, and end stage renal disease. •Black patients had lower rates of COPD and asthma	Mortality	•Among the hospitalized black adults, 22.7% died, among hospitalized white adults, 20.8% ($p = 0.013$)
**55**	Ghoneim, S. (2020) [34]	USA	Retrospective analysis	61493400	•African, •Non-african	•Hypertension •Diabetes mellitus •Obesity •Dyslipidemia	•African americans are more likely to have hypertension, obesity, or diabetes	Mortality	•In Chicago, 70% of all COVID-19 deaths were seen in African Americans, who only represent 29% of the city's population.

**56**	Luck, A. N. (2022) [35]	USA	Retrospective study	225,006 and 226,635	•Non-Hispanic White •Non-Hispanic Black •Hispanic	•Alzheimer's •cerebrovascular diseases, •Diabetes mellitus •Diseases of heart, •Influenza •Pneumonia Chronic lower respiratory disease •Nephritis	•Hispanic individuals have a higher prevalence of diabetes and heart disease	Mortality	•Hispanic COVID-19 death rate in 2020 was ~1.1 times higher than the Black death rate and ~2.3 times higher than the White death rate.
**57**	Wong, M. S. (2021) [36]	USA	Retrospective study	705,715	•Asian •non-Hispanic Black •Hispanic •non-Hispanic White •NHOPI •American Indian/American Native	•Asthma •liver disease •obesity •diabetes •heart disease •immunocompromisation •chronic pulmonary disease •chronic kidney disease	•Non-Hispanic Whites had the smallest proportion of asthma and chronic kidney disease. •Asians had the smallest proportion with chronic pulmonary disease, type 2 diabetes, heart disease, immunocompromisation, liver disease, and obesity. •Non-Hispanic Blacks were most likely to have chronic kidney disease, type 2 diabetes, or be immunocompromised. •Hispanics were more likely to have liveer disease	Mortality	Mortality was observed over time •Initial, non-Hispanic black (12%), Hispanic white (10.2%) •Adjusted case fatality from spring - summer, American Indian/American Native (10.7%), Asian (10.5%), Hispanic (7.2%), non-Hispanic white (5.7%) •Further adjusted case fatality rate form summer - fall, non-Hispanic black (1.9%), non-Hispanic White (2.5%), other groups (2.4-3.3%)
**58**	Joynt Maddox, K. E. (2022) [76]	USA (Missouri)	Retrospective study	73635	•Non-Hispanic Black •Non-Hispanic White •Hispanic	•Psychiatric Disorder •Alzheimer's/Dementia •Asthma •Chronic Kidney DIsease •COPD •Heart Failure •Hypertension	•NH White patients were the most likely to have evidence of a psychiatric disorder. •NH White patients were less likely to have asthma, chronic kidney disease, heart failure, hypertension, obesity, tobacco use, or uncontrolled diabetes	Mortality	•NH Black 58.3 per 100 000, NH other/unknown 38.9 per 100 000, NH White 19.4 per 100 000, Hispanic 14.8 per 100 000

						•Obesity •Diabetes	compared to NH Black Patients.		
**59**	Hidalgo, D. C. (2022) [77]	USA (Illinois)	Cross-sectional cohort study	257	•Non-Hispanic Black •Non-Hispanic White •Hispanic	•Hypertension •Chronic kidney disease •End-stage renal disease •Heart failure •Coronary artery disease •Chronic lung disease	•Non-Caucasian Hispanic patients had a lower prevalence of hypertension, chronic kidney disease, end-stage renal disease, heart failure, coronary artery disease, and chronic lung disease compared to non-Hispanics	Mortality	•The overall 30-day mortality rate was 17.1% (n=44); 11.9% in the Hispanic group vs 26.3% in the non-Hispanic group

Supplementary Table 1. Data extraction table for the review.

Study #	First Author	Country	Type of Study	Sample Size	Study Population	Ethnicity Group(s) Examined	Sociodemographic Factors Examined	Sociodemographic differences
**1**	Wiley, Z. (2021) [51]	USA (Atlanta)	Retrospective cohort study	1. 831 Total 2. 569 Black (68.5%) 3. 32 Hispanic (3.9%)	Adults 18-49, 50-64, 65+	1. Black 2. Non-Black	1. Insurance 2. Age	1. 96.7% had healthcare insurance (Black patients) 2. 93.9% had healthcare insurance (non-Black patients) 3. In comparison to non-Black patients, the Black patients were younger
**2**	Datta, B. K. (2022) [19]	USA	Retrospective analytical model		Adults 18+	1. Black 2. White 3. Hispanic 4. Other	1. Age	1. Older age groups had greater rate changes in ICU admissions – ages 65+

**3**	Salvatore, M. (2021) [37]	USA (Michigan)	Association Study	53,853		1. Non-Hispanic Black/African American (Black)<br>2. Non-Hispanic White (White)	N/A	1. N/A<br>2. (Although, it does say that "models are adjusted for age, sex, race, and three census tract-level socioeconomic indicators: proportion with less than high school education, proportion unemployed, and proportion with annual income below the federal poverty level".) It does not elaborate on those points though.
**4**	Millett, G. A. (2020) [20]	US counties		N/A	<65 years old, >65 years old	1. Black<br>2. White	1. Insurance<br>2. Employment	1. The PAF for lack of health insurance was 3.3% for counties with less than 13% black residents and 4.2% for counties with greater than or equal to 13% black residents.<br>2. The protective effect of unemployment was also larger for counties with higher proportions of black residents. However, the paper stated that "None of these determinants were associated with significant excess of COVID-19 deaths"
**5**	Olanipekun, T. (2022) [52]	USA (Atlanta, Georgia)	Retrospective Cohort Study	181	Adults 18+	1. Black<br>2. White<br>3. Hispanic	1. Sex<br>2. BMI<br>3. Age	1. Mortality rates were higher in males (59.03% vs 50.8%, P = 0.03), in patients with BMI ≥ 35 kg/m2 (43.4% vs. 32.2%, p < 0.01).<br>2. Age was also a predictor, (64 years IQR, 62-66 vs.52 years IQR, 52-56 years, p < 0.01), in the 55-64-year range (44.1% vs. 27%, p < 0.01), and > 75 years of age (20.3% vs. 12.3% P =0.04).
**6**	Suresh, S. (2021) [38]		Retrospective study	1,983		1. African American	1. Sex<br>2. Race<br>3. Age	1. Patients with obesity were younger (58.2 years vs. 69.9 years), higher proportion were females (55% women vs. 45% men) and African American (63% vs. 49%) in comparison to those who were not obese

**7**	Hehar, J. (2022) [39]	USA (Detroit, Michigan)	Retrospective cohort study	8,751	Adults 18+	1.Black<br>2. White	1. Sex<br>2. Age	1. According to the CDC, with increasing age comes an increased risk of hospitalization and death even beginning at the age of 18 years old.<br>2. There is a significant increase in risk in the 65- to 74-year age groups<br>3. The highest risk was found to be in the ≥85 years age group with up to 8700-fold increase in risk<br>4. Data showed that there is an increase of risk of higher mortality and ICU admissions per age year<br>5. Male sex is associated with both outcomes (mortality and ICU admissions)
**8**	Kabarriti, R. (2020) [45]	USA (New York)	Cohort Study	9,268	Adults	1. Non-Hispanic Black<br>2. Non-Hispanic White<br>3. Hispanic<br>4. Asian<br>5. Other	1. Sex<br>2. Socioeconomic status<br>3. Age	1. Poor outcomes from COVID-19 are significantly associated with the following comorbidities: obesity, cardiovascular disease, diabetes, kidney disease, and dementia.<br>2. Looking at non-Hispanic Black patients, those groups that were the male sex, those who had a BMI greater the 35, diabetes, kidney disease and dementia were significantly associated with an increased risk of mortality.
**9**	Yehia, B. R. (2020) [21]	USA	Cohort Study	11,210	18+	1. Black<br>2. White<br>3. Other	1. Sex<br>2. Insurance status<br>3. Age<br>4. Neighborhood deprivation<br>5. Site of care	1. The median age in this study was 61 years.<br>2. Black patients were younger in comparison to White patients (66 vs 61 years)<br>3. Black patients were more likely to be female (49% vs 54%)<br>4. Black patients were more likely to have Medicaid insurance (13.3% vs 24.7%)<br>5. Black patients had higher median scores on the Neighborhood Deprivation Index and the Elixhauser Comorbidity Index)

**10**	Best, J. H. (2020) [22]	USA	Retrospective, observational study	3,471	Adults	1. African American<br>2. White	1. Sex<br>2. Race<br>3. Age<br>4. Geographic region	1. African American patients found to be younger in comparison to White patients.<br>2. Higher rates of these patients were females in comparison to White patients.
**11**	Alkhatib, A. L. (2020) [23]	USA	retrospective cross-sectional analysis	158	Adults	1. African American	1. Age<br>2. BMI	1. Age, greater BMI, and obstructive lung disease were associated with more cases of severe COVID-19 infections in African American patients.
**12**	Yu, Y. (2021) [40]	USA (Michigan)	Retrospective cohort study	249,075		1. Black<br>2. White	1. age<br>2. self-reported sex<br>3. race/ethnicity<br>4. smoking status<br>5. alcohol consumption<br>6. body mass index (BMI)<br>7. Neighbourhood Socioeconomic Disadvantage Index (NDI) [22]<br>8. population density (persons per square mile)	1. Overall, younger patients, female sex, higher BMI, being an ever smoker, alcohol consumption, Black race, lower NDI, areas with higher population densities, and accumulation of comorbidities were associated with an increased chance of getting tested
**13**	Dai, C. L. (2021) [59]	USA (California, Oregon, and Washington)	Retrospective cohort study	629,953		1. non-Hispanic White<br>2. Hispanic<br>3. non-Hispanic Asian American<br>4. non-Hispanic Black<br>5. non-Hispanic American Indian/Alaska Native<br>6. non-Hispanic	Age	1. Hispanic patients tested positive at a higher rate, required excess hospitalization and mechanical ventilation, and had higher odds of in-hospital mortality despite younger age<br>2. Compared with White patients, mean ages were lower among patients of minority race/ethnicity, except for Asians, who had a similar mean age<br>3. Charlson Comorbidity Index scores were also lower among minority patients

						Native Hawaiian/Pacific Islander<br>7. non-Hispanic other<br>8. unknown		
**14**	Kalyanaraman M. R. (2020) [46]	USA (New York)		22,254	Adults	1. Black<br>2. White<br>3. Hispanic	1. Sex<br>2. Age<br>3. BMI	1. Male sex, older age, diabetes, cardiac history, and chronic kidney disease were significantly associated with testing positive, hospitalization, and death<br>2. Racial/ethnic disparities were observed across all outcomes
**15**	Izzy, S. (2020) [60]	USA (Massachusetts)		5,190	18+	1. White<br>2. Latinx<br>3. African American<br>4. Asian American	1. Age<br>2. Gender	1. More Latinx and African American patients in the younger age groups were hospitalized than White patients<br>2. Within each ethnic and racial group, age and male gender were independently predictive of hospitalization
**16**	Gu, T. (2020) [41]	USA (Michigan)	Cohort Study	5,698		1. Non-Hispanic White<br>2. Non-Hispanic Black	1. Age<br>2. Sex<br>3. Population density<br>4. Socioeconomic status	1. High population density, type 2 diabetes, and kidney disease were associated with hospitalization, in addition to older age, male sex, and obesity<br>2. Older age, male sex, obesity, and living in densely populated areas was associated with increased risk of hospitalization
**17**	Parker, J. J. (2021) [61]	USA (Tennessee)		130,040	Categorized into 3 groups: alive (living) case-patients, standard surveillance COVID-19 deaths	1. White was defined as White race, non-Hispanic ethnicity<br>2. Black as Black race, non-Hispanic ethnicity<br>3. Hispanic was defined as all races that selected Hispanic ethnicity	1. Age<br>2. Sex	The mean age of living and deceased patients also differed by race; for deceased patients the average age was 75.6 years for White patients, 69.5 years for Black patients, and 61.3 years for Hispanic patients. Patients who died from COVID-19 were more likely to be older, male, and Black and to have underlying conditions (hereafter comorbidities) than case-patients who survived.

**18**	Lobelo, F. (2021) [53]	USA (Atlanta)	Retrospective cohort study	5,712		1. Black<br>2. White<br>3. Hispanic<br>4. Asian<br>5. Other	1. Age<br>2. Sex<br>3. Residence location/ZIP code<br>4. Household income<br>5. Insurance<br>6. Occupation<br>7. Education	1. Male patients had higher rates of ICU admissions compared to female patients<br>2. Older patients (aged 50-64, 65+) had higher rates of ICU admission and death<br>3. Black patients had the highest proportions of living in neighbourhoods under the federal poverty line and in more deprived locations
**19**	Marmarchi, F. (2021) [54]	USA (Atlanta, Georgia)	Cohort study	288	Adults 18+	1. African Americans	1. Race<br>2. Age<br>3. Gender	1. Race was not associated with hospital mortality after adjusting for differences in sociodemographic and clinical characteristics<br>2. Factors associated with in-hospital mortality included age but not race
**20**	Kolinski, J. M. (2020) [62]	USA (Wisconsin)	Retrospective analysis	168	Adults 18+	1. African American<br>2. White<br>3. Other	1. Age<br>2. Sex<br>3. Location prior to admission	1. 55% of admissions were male
**21**	Casillas, E. (2021) [55]	USA	Retrospective cross-sectional study	278		1. Hispanic<br>2. Non-Hispanic	1. Age<br>2. ZIP code<br>3. Insurance	1. Patients who identified as Hispanic were younger
**22**	Musshafen, L. A. (2022) [64]	USA (Mississippi)	Retrospective cohort study	1,230	Adults 18+	1. American Indian/Mississippi Band Choctaw Indian<br>2. African American/Black<br>3. Caucasian/White<br>4. Asian or Native Hawaiian/Pacific Islander<br>5. Other	1. Age<br>2. Sex<br>3. Race	1. Age, race, and sex were statistically significant predictors of inpatient mortality in the total inpatient population

**23**	Lopez, D. C. (2022) [65]	USA (Cleveland)	Retrospective cohort study	2,125	Adults 18+	1. Black<br>2. White<br>3. Other, which includes Asian, Native Hawaiian, Pacific Islander, American Indian, Alaskan Native, multiracial, non-Hispanic, and Hispanic	1. Race/ethnicity	1. No difference in mortality or length of stay outcomes by race or ethnicity.<br>2. Among the 2108 patients included in the race analysis, 41.8% were female, 33% identified as Black, and 6.7% identified as being of non-White or non-Black race<br>3. The Black cohort was younger than the White cohort
**24**	Zakaria, A. (2021) [42]	USA (Michigan)	Retrospective observational study	1,064	18+	1. African American<br>2. Non-Hispanic White	1. Age<br>2. Race<br>3. Gender	1. AA were significantly younger compared to WH (mean age 64.4 ± 16.7 vs. 68.1 ± 19.1), which was observed in both sexes<br>2. There were more females among the AA patients than WH patients (53.4% vs. 45.7%)
**25**	Shaw, P. A. (2022) [66]	USA (Pennsylvania)	Retrospective	6,953	Adults	1. Black<br>2. White<br>3. Hispanic/Latinx	1. Age<br>2. Race	1. Younger patients (< 40 years) were the least common group in March-April 2020 but the most common group in July-October 2020 to be hospitalized, then returned to being the least common group in January-March 2021<br>2. 51.5% of admitted patients in March-April 2020 identified as Black compared to only 35.0% in January-March 2021
**26**	Kodsup, P. (2022) [67]	USA (Louisiana)	Negative binomial regression analyses	408,047	Adults	1. African American<br>2. White American	1. Age<br>2. Race	1. Age was significantly correlated with increased numbers of COVID-19 mortality and incidence<br>2. African American COVID-19 death was three times higher than other races

**27**	Abate, G. (2022) [69]	USA (Midwest region)	Retrospective study	N/A	Adults 18+	1. African Americans<br>2. Caucasians	1. Age<br>2. Sex<br>3. Insurance	1. Patients who were not African American or Caucasian were of younger age (mean 57.3 years)<br>2. Blacks had the lowest rate of health insurance and least access to private health care
**28**	Shadyab, A. H. (2021) [56]	USA (California)	Retrospective cohort study	100	65+	1. Hispanic (46%)<br>2. Non-Hispanic (4.0% White, 10.0% Black, 6.0% Asian, 39.0% other race or mixed race)	1. Age<br>2. Sex<br>3. Race	1. The rate of hospitalization or death was not significantly different between Hispanic and non-Hispanic patients with COVID-19
**29**	Cervantes, J. (2021) [70]	USA (Mexico border)		82		1. Hispanic	1. Age<br>2. Sex	1. No demographic characteristics were associated with any outcomes except age
**30**	Ricardo, A. C. (2022) [24]	USA	Multicenter Cohort Study	2,153	46+	1. Hispanic (46.2%)<br>2. Non-Hispanic White (53.8%)	1. Sex	1. Males had a higher % of 28-day mortality (689 male vs 305 female (Hispanic) and 773 male vs 386 female (White))

**31**	Price-Haywood, E. G. (2020) [68]	Louisiana	Retrospective cohort study	3,481		1. Black non-Hispanic (70.4%)<br>2. White non-Hispanic (29.6%)	1. Age<br>2. Sex<br>3. Race<br>4. Insurance	1. Increasing age, public insurance (Medicare or Medicaid), and residence in a low-income area were associated with increased odds of hospital admission<br>2. Female sex was associated with lower odds of admission
**32**	Xie, J. (2020) [71]	New Orleans	Retrospective, observational study	287		1. Black/African American	1. Age<br>2. Sex	1. Mean age was 61.5 years<br>2. 56.8% were female<br>3. 85.4% were non-Hispanic Black
**33**	Page-Wilson, G. (2021) [47]	New York City	Retrospective cohort study	928	Median age was 64 years (IQR 52-75)	1. Black<br>2. Asian	1. Race<br>2. BMI<br>3. Distressed community index	1. Race, ethnicity, and socioeconomic status did not impact COVID-19 related mortality
**34**	Raiker, R. (2021) [25]	USA	Comparative cohort study	9,730 with rheumatoid arthritis; 656,979 without rheumatoid arthritis	18 years of age or older	1. White<br>2. Black<br>3. Asian	1. Age<br>2. Sex<br>3. Race<br>4. BMI	1. Male sex, black race, and glucocorticoid use increased the risk of adverse outcomes

**35**	Nguyen, J. L. (2022) [26]	USA	Retrospective population-based cohort study	679,566	Adults	1. Black/African American	1. Age<br>2. Sex<br>3. Race	1. Older age was associated with higher population attributable factors<br>2. Population attributable factors for cardiovascular disease and diabetes were generally higher for female sex compared with male sex, especially among patients aged 50–64 years
**36**	Arasteh, K. (2020) [27]	USA		10,076	Adult, 18-44,45-64,65+	1. Black<br>2. Hispanic<br>3. White	1. Occupation<br>2. Poverty	1. Hispanics and Blacks in NYC are overrepresented among frontline and essential workers, including among healthcare workers, police, firefighter, grocery clerks, and others who are at a heightened risk of potential exposure to the virus<br>2. Neighborhood poverty compounded the relative risk of diabetes of Blacks and Hispanics in the ages 18 - 44 and 65 or older compared with Whites
**37**	Racine, R. (2022) [72]	USA (Southwest Georgia)	Retrospective cohort study	710	Adult, >=18	1. African American<br>2. Non-African American (Caucasian, Asian, other race)	n/a	1. African Americans do not demonstrate an increase in mortality or other outcomes such as need for mechanical ventilation, ICU care, and new dialysis<br>2. African Americans show an increased risk in COVID-19 related hospitalizations<br>3. In-hospital mortality was similar between the groups, as was need for mechanical ventilation, ICU care, and new dialysis

**38**	Wiley, Z. (2022) [28]	USA	Cohort study	94,683	Adult, >=18	1. Hispanic<br>2. Black<br>3. White	1. Age<br>2. Sex<br>3. Insurance	1. Older age and male sex each had increased relative risk of in hospital mortality<br>2. Higher proportion of uninsured patients in the emergency department cohort compared with the hospitalized cohort<br>3. There were racial/ethnic differences in the distribution of health insurance: 43% of Hispanics, 68.5% of Blacks and 70% of Whites were insured<br>4. For patients in the emergency department, the proportion of insured patients with Medicare was 33.4% for White patients, 24.5% of Black patients and 9.4% of Hispanic patients<br>5. Proportion of uninsured status highest in Hispanic patients, with 16.9% without insurance, followed by 12.8% without insurance for Black patients and 8.2% for White patients
**39**	Rodriguez, F. (2020) [29]	USA		8,590	>=18 years old	1. Non-Hispanic White<br>2. Non-Hispanic Black<br>3. Hispanic (All races)<br>4. Asian / Pacific Islander	1. Age<br>2. Insurance<br>3. Education<br>4. Income / Employment	1. Hospitalized Hispanic and Black patients were substantially younger than non-Hispanic White patients (median age of 57 and 60 vs 69 respectively)<br>2. Hispanic patients were more than twice as likely to be uninsured than other groups and had the lowest education attainment<br>3. Black patients had the lowest median household income and highest unemployment rate
**40**	Elbadawi, A. (2022) [73]	USA		3,678	Adults, >=18	1. Caucasian<br>2. Black<br>3. Hispanic<br>4. Other	1. Age<br>2. Sex	1. Among racial groups, Caucasian patients were generally older, followed by Hispanic patients, Black patients, and other races<br>2. Hospitalization (≥75 years) was more frequent among Caucasians (34.6%), followed by Black patients (25.2%), Hispanic patients (20.7%), then other racial groups (12.2%)<br>3. Among female patients hospitalized with COVID-19, the majority

								were Black (51.9%), followed by Caucasians (43.9%), Hispanics (41.4%), then other racial groups (29.3%)
**41**	Metra, B. (2021) [30]	USA	Retrospective observational cohort study	346,953	Adults, >=18	1. Non-Hispanic Black<br>2. Non-Hispanic White	1. Age<br>2. Sex<br>3. Region	1. Blacks had a higher prevalence of homelessness
**42**	Krishnamoorthy, G. (2020) [43]	USA	Retrospective observational study	799		1. Black<br>2. White	1. Age<br>2. BMI<br>3. Smoking status	1. White patients were on average older as compared with Black patients (71.8 vs. 62.9 years, P < .032) and presented to the hospital a day earlier (5.4 vs. 6.6 days, P < .006)<br>2. Statistically significant differences between the two groups in regard to BMI, smoking status, and diabetes as comorbid condition

**43**	Parpia, A. S. (2021) [44]	USA (Michigan)		6,065		1. White<br>2. Black<br>3. Native American<br>4. South Asian<br>5. Southeast Asian<br>6. East Asian<br>7. other Asian<br>8. Hispanic/Latinx<br>9. Non-Hispanic/Latinx<br>10. Other/not reported	1. Age<br>2 - Sex<br>3 - Employment status<br>4 - High-risk or congregate living facility exposure as resident or staff	1. Black individuals who died from COVID-19 were significantly younger (median [IQR]: 72 [63, 81], p<0.001) than White individuals (81 [72, 89]) and reported lower rates of being to be retired or unemployed (12.9%, p<0.001) than White decedents (22.2%)<br>2. Among all COVID-19 deaths, 44.0% were either residents or employees of high-risk or congregate living facilities, such as long-term care and senior homes, homeless shelters, and prisons<br>3. Black decedents (29.7%) had lower rates of living or working in high-risk or congregate living facilities than White individuals (54.8%, p<0.001)<br>4. Among all decedents who worked or resided in these facilities, 90.6% were aged 65 and older
**44**	Escobar, G. J. (2021) [57]	USA (Northern California)	Retrospective cohort study	3,481,716	Adults, >=18	1. White<br>2. African American<br>3. Hispanic<br>4. Asian<br>5. Other/unknown	1. Age<br>2. Sex<br>3. Neighbourhood deprivation index	1. Likelihood of infection was largely due to race (80.3%)<br>2. For other outcomes, age was most important; race only contributed 4.5% for hospitalization, 12.8% for admission illness severity, 2.3% for in-hospital death, and 0.4% for any death<br>3. White persons and Asian persons lived in more advantaged neighborhoods, as evidenced by lower NDI, than did Black/African American persons, Hispanic persons, and persons of other/unknown races/ethnicities<br>4 - Hospitalization, ICU admission, assisted ventilation, in-hospital death, and total deaths among infected patients during the study period were higher among African American persons, Hispanic persons, and Asian persons

**45**	Muñoz-Price, L. S. (2020) [63]	USA (Wisconsin)	Cross-sectional study	369	Adults (18 years and older) who are not health system employees	1. Black<br>2. White<br>3. Native Hawaiian,<br>4. Native American or Alaska Native<br>5. Asian<br>6. Unknown	1. Age<br>2. BMI<br>3. Poverty	1. Older age (≥60 years: OR, 22.79; 95% CI, 3.38-53.81; P = .001) significantly increased the likelihood of mortality<br>2. Higher BMI was also significantly associated with mortality, each BMI unit increasing the odds of mortality by 19% (OR, 1.19; 95% CI, 1.05-1.35; P = .006)<br>3. Poverty increased by more than 3-fold the odds of ICU admission (OR, 3.58; 95% CI, 1.08-11.80, P = .04)
**46**	Egede, L. E. (2020) [74]	USA (Milwaukee and Southeast Wisconsin)	Cross-sectional analysis	31,549	Adults	1. 4.5 percent non-Hispanic Whites<br>2. 14.9 percent non-Hispanic Blacks<br>3. 14.8 percent Hispanics	1. Sex<br>2. Age<br>3. Location<br>4. Primary payor<br>5. Tobacco use<br>6. BMI	1. Men were more likely to screen positive (OR, 1.28; 95% CI, 1.16, 1.41) than women<br>2. Self-pay/uninsured individuals were more likely to screen positive compared with those with a managed care/commercial payor (OR, 1.34; 95% CI, 1.09, 1.64)<br>3. Individuals living in Milwaukee County and the counties immediately surrounding it were more likely to screen positive compared with those in other counties that have primarily rural, White populations (OR, 2.21; 95% CI, 1.64, 2.99)
**47**	Bushman, D. (2021) [48]	USA	Case-control study	15,097	21 - 64 years old	1. Non-Hispanic Asian<br>2. Non-Hispanic Black<br>3. Hispanic/Latino<br>4. Non-Hispanic White<br>5. Non-Hispanic Other	1. Sex<br>2. Race	1. Patients predominantly male (65.5%), Hispanic (37.6%) or Black (29.1%), and residents of Queens (37.1%) or the Bronx (29.8%), and resided in medium poverty neighborhoods (37.0%).<br>2. Black and Hispanic populations in NYC are at higher risk of COVID-19 compared with other racial and ethnic groups.<br>3. COVID-19 mortality rates in NYC are highest among Black and Hispanic persons.<br>4. Race/ethnicity was not independently associated with mortality among study patients, yet racial/ethnic disparities in deaths were observed in NYC

**48**	Shakil, S. S. (2022) [31]	USA	Retrospective Cohort Study	21,073	18 - 30, 31 - 40, 41 - 50, 51 - 60, 61 - 70, 71 - 80, 81 - 90, 91 - 110	1. Hispanic 2. Non-Hispanic Black 3. Non-Hispanic White 4. Asian 5. Other	1. Age 2. Sex 3. Race 4. Ethnicity	n/a
**49**	Toth, A. T. (2021) [49]	USA	Retrospective Cohort Analysis	2,233	18 - 44 years old. 45 - 64 years old. 65 - 74 years old. 75 and older	1. Hispanic 2. Non-Hispanic Black/African American 3. Non-Hispanic White 4. Non-Hispanic Asian/Pacific islander 5. Non-Hispanic other	1. Poverty 2. Insurance 3. Age	1. Patients from medium, high, and very high poverty neighborhoods showed significant reductions in mortality from high/very high to low/medium 2. Primary payer (uninsured vs commercial/other) was associated with higher odds of death; patients on Medicaid and those uninsured had higher odds ratios for mortality compared with insured patients 3. Age was associated with higher odds of death
**50**	Ashktorab, H. (2022) [32]	USA	Retrospective Study	5,852	<35 years old, 35 - 44 years old, 45 - 54 years old, 55 - 64 years old, 65 - 74 years old, >=75 years old	1. African American 2. European American 3. Latin American 4. Asian American	1. Age 2. Sex	1. Age was a very strong predictor of mortality; compared to individuals < 35 years old, patients >= 75 year old were at a higher risk of death 2. Male sex was a predictor of mortality

**51**	Ebinger, J. E. (2021) [58]	USA	Retrospective Cohort Study	1,584	Mean age was 61.58	1. Asian<br>2. Hispanic/Latinx<br>3. Non-Hispanic Black<br>4. Non-Hispanic White	1. Age<br>2. Sex<br>3. Race and ethnicity	1. Hispanic/Latinx compared with non-Hispanic White patients had higher odds of experiencing more severe illness among hospitalized patients (OR 2.28, 95% CI 1.62 to 3.22), with this disparity persisting over time<br>2. During the initial 2 months of the pandemic, non-Hispanic Blacks were more likely to suffer severe illness than non-Hispanic Whites (OR 2.02, 95% CI 1.07 to 3.78); this disparity improved by May, only to return later in the pandemic<br>3. Compared with non-Hispanic Whites, Hispanic/Latinx and non-Hispanic Black patients tended to be younger<br>4. Hispanic/Latinx patients hospitalized for COVID-19 were consistently at the highest risk for the most severe forms of COVID-19 illness compared with non-Hispanic White patients<br>5. Hispanic/Latinx patients have greater risk for more severe forms of COVID-19 illness even while being younger and with fewer comorbid conditions compared to non-Hispanic White patients
**52**	Golestaneh, L. (2020) [50]	USA	Retrospective Cohort Study	505,992	<=40 years old, 41 - 50 years old, 51 - 60 years old, 61 - 70 years old, 71 - 80 years old	1. NH White<br>2. NH Black<br>3. Hispanic<br>4. Other	1. Poverty<br>2. Age	1. Black patients more frequently lived in communities with a higher percentage of Black residents and households living below the poverty line<br>2. Age category was significantly associated with hospitalization
**53**	Samuels, S. (2021) [75]	USA	Retrospective Analysis	1,692	3 groups: pediatric (0 - 17 years old), non-elderly (18-64 years old) and elderly (65	1. NH Black<br>2. NH White<br>3. Hispanic	1. Age	1. Average age of confirmed cases among patients who were admitted to the ICU was 51 years old

					years and older)			
**54**	Navar, A. M. (2021) [33]	USA	Retrospective Cohort Study	19,584	Adults	1. Black or African American<br>2. Hispanic<br>3. White<br>4. American Indian or Alaska Native<br>5. Asian or Pacific Islander<br>6. Mixed racial group	1. Age<br>2. Sex<br>3. Ethnicity	1. Hispanic ethnicity was associated with lower mortality than non-Hispanic patients<br>2. Mortality was higher among Black patients (relative to White patients) after adjusting for demographic factors including age, sex, date, region, and insurance status
**55**	Ghoneim, S. (2020) [34]	USA	Retrospective analysis	61,493,400	Adults	1. African<br>2. Non-African	1. Sex<br>2. Race<br>3. Occupation<br>4. Poverty	1. Cumulative incidence of COVID-19 was higher in women than in men (60% vs 40%)<br>2. Across all countries, prevalence studies have shown men with COVID-19 are at more risk of worse outcomes and death<br>3. African Americans are more likely to carry out high-risk essential jobs, including low-wage jobs that cannot be done remotely<br>4. African Americans are more likely to be uninsured, more segregated, and in concentrated poverty

**56**	Luck, A. N. (2022) [35]	USA		225,006 and 226,635	Adults 25+ in 10-year age groups	1. Non-Hispanic White<br>2. Non-Hispanic Black<br>3. Hispanic	1. Race<br>2. Residence<br>3. Health insurance 4. Finances	1. Hispanic community are at a disadvantage due to occupational, residential, and socioeconomic conditions<br>2. Hispanic individuals are disproportionately represented in essential occupations with high exposure to COVID-19 and limited workplace protections<br>3. Hispanic populations are more likely to live in denser, multigenerational households<br>4. Hispanic communities have the lowest rate of health insurance coverage of any racial/ethnic group in the US (20% uninsured)
**57**	Wong, M. S. (2021) [36]	USA		705,715	Adults	1. Asian<br>2. Non-Hispanic Black<br>3. Hispanic<br>4. Non-Hispanic White<br>5. Native Hawaiian or other Pacific Islander<br>6. American Indian/America n Native	1. Race<br>2. Poverty	1. American Native/Indian experience systemic racism, historical and political oppression, and violence, which leads tofacing unequal social determinants such as poverty and substandard housing conditions.
**58**	Joynt Maddox, K. E. (2022) [76]	USA	Patient level analysis	73,635		1. Non-Hispanic Black<br>2. Non-Hispanic White<br>3. Hispanic	1. Race	1. Black and Hispanic individuals tend to receive care in lower-quality hospitals<br>2. Access to testing facilities also differs by race, more cases can go unconfirmed in communities of color<br>3. Black and Hispanic individuals, as well as those in other groups are more likely to be essential workers and to be in high contact essential employment positions (health care, education and public transport)<br>4. Non-Hispanic Black and Hispanic individuals on average live in more densely populated ZIP codes and in denser housing than non-Hispanic White individuals

**59**	Hidalgo, D. C. (2022) [77]	USA (Illinois)	Cross-sectional cohort study	257	Adults 18+	1. Non-Hispanic Black<br>2. Non-Hispanic White<br>3. Hispanic<br>4. Asian	1. Age<br>2. Race	1 - The median age at admission of Hispanic patients was significantly lower compared to non-Hispanic patients (56.6 vs. 65.7 years, p<0.01)

Supplementary Table 2. Data extraction table including socio-demographic variables.
